# Great Offset Difference Internuclear Selective Transfer

**DOI:** 10.1021/acs.jpclett.3c00194

**Published:** 2023-04-20

**Authors:** Evgeny Nimerovsky, Eszter Éva Najbauer, Stefan Becker, Loren B. Andreas

**Affiliations:** Department of NMR-based Structural Biology, Max Planck Institute for Multidisciplinary Sciences, Am Fassberg 11, Göttingen 37077, Germany

## Abstract

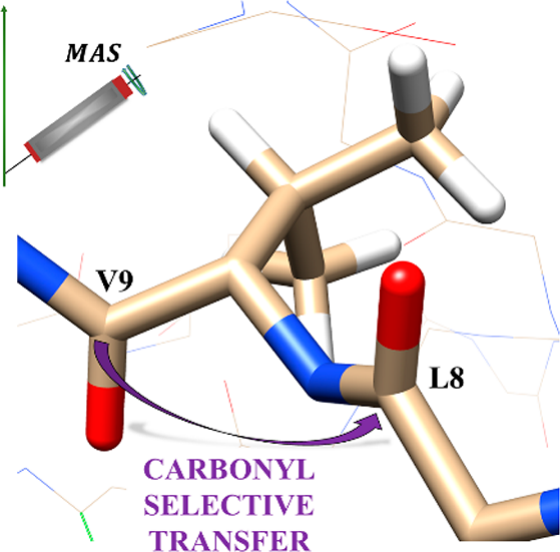

Carbon–carbon dipolar recoupling sequences are frequently used building blocks in routine magic-angle spinning NMR experiments. While broadband homonuclear first-order dipolar recoupling sequences mainly excite intra-residue correlations, selective methods can detect inter-residue transfers and long-range correlations. Here, we present the great offset difference internuclear selective transfer (GODIST) pulse sequence optimized for selective carbonyl or aliphatic recoupling at fast magic-angle spinning, here, 55 kHz. We observe a 3- to 5-fold increase in intensities compared with broadband RFDR recoupling for perdeuterated microcrystalline SH3 and for the membrane protein influenza A M2 in lipid bilayers. In 3D (H)COCO(N)H and (H)CO(CO)NH spectra, inter-residue carbonyl–carbonyl correlations up to about 5 Å are observed in uniformly ^13^C-labeled proteins.

Dipolar recoupling elements^1−4^ are the building blocks of various multidimensional proton-detected magic-angle spinning (MAS) NMR experiments^5−9^ that are crucial in both structure determination and in exploration of the dynamics of biological macromolecules.^10−19^ Homonuclear carbon–carbon dipolar recoupling sequences^20−31^ are crucial in amino-acid-typing during sequential assignment,^32−34^ as well as for distance measurements.^35−37^

Dipolar recoupling sequences are characterized as either first-order or second-order sequences.^1,3^ Transfer between isolated two-spin systems can be observed with first-order sequences, since they recouple two-spin terms in the Hamiltonian, such as the dipolar coupling. For second-order sequences, the transfer dynamics involve at least three spins, since the relevant terms in the recoupled Hamiltonian depend on two dipolar couplings among three spins. First-order sequences have the potential advantage of relatively straightforward analysis, as the transfer depends on only two spins for isolated spin pairs.^38^ However, broadband first-order carbon–carbon recoupling applied to uniformly labeled proteins is subject to dipolar truncation,^39^ such that mostly intra-residue cross-peaks are observed. Second-order recoupling sequences,^1,3,4,11,40^ based on proton-driven spin diffusion^41−47^ or third spin assistance,^48,49^ can reduce the influence of dipolar truncation. While these methods result in increased intensities for long-distance correlations, the relationship between peak intensity and distance is less straightforward than in the case of first-order recoupling sequences since they depend on an additional spin interaction.

Selective methods have also been developed to overcome the aforementioned problems in order to more effectively measure weaker, long-distance inter-residue correlations critical for structure determination. Specific spin-labeling provides an alternative solution^50−54^ that at the same time can yield exquisite line widths. Selective recoupling experiments^11^ based on band-selective pulses,^55^ effective rf-field power matches,^56,57^ zero-^58,59^ or double-quantum^60^ shift evolution, symmetry rules,^61,62^ phase-optimization,^63−65^ and optimal control algorithms^66^ have been developed. For fast MAS (∼55 kHz and above), both double-quantum (DQ) as well as zero-quantum (ZQ) methods have been developed. DQ sequences are characterized by a Hamiltonian that induces simultaneous spin flips, while for ZQ sequences, spin flip-flops (no change in total spin angular momentum) are induced. Double-quantum sequences^56,57,60−65,78^ are not ideal for the detection of correlations that correspond to longer distances in uniformly labeled proteins, since relayed transfer^57^ can cancel direct transfer. They have, however, been successfully applied for quantitative distance measurement.^67^ MODIST,^68^ a selective method developed for proton recoupling, did not efficiently recouple ^13^C (Figure S7A). We therefore sought a new zero-quantum (ZQ) pulse sequence that achieves *ẑ*–*ẑ* mixing with limited relaxation loss and is efficient only for spins with similar chemical shifts (e.g. among carbonyl or aliphatic spins).

Here, we present a first-order zero quantum recoupled method, the Great Offset Difference Internuclear Spin Transfer (GODIST) pulse sequence, which allows selective observation of aliphatic–aliphatic and carbonyl–carbonyl correlations at fast and ultra-fast MAS rates. Starting from the MODIST sequence^68^ we modified both the flip angles and the phase cycling to achieve a selective and efficient transfer between carbons of similar chemical shifts. Using numerical simulations, we restricted our simulated search space to 1–32 rotor periods, 0.125–16 pulses per rotor period, flip angles of 12.5°–360°, and phase steps of 90°. We optimized for minimal transfer between carbonyl and aliphatic spins, maximal transfer between carbonyl spins, maximal retention of the initial carbonyl signal (sum of the remaining and transferred signals), and a width of 6–7 kHz for the selective transfer (broad enough to cover the desired region).

Figure 1 shows simulations of the optimized sequence, GODIST, for a two-spin system (two carbonyl spins), as well as a four-spin system (two carbonyl and two aliphatic spins). The GODIST sequence consists of 32 2π pulses with a *yy̅xx̅y̅yxx̅yy̅xx̅yy̅x̅xy̅yx̅xyy̅x̅xy̅yx̅xy̅yxx̅* phase cycle (Figure 1A). The total length of the sequence is 64 rotor periods, which results in a carbon rf nutation frequency of half the MAS rate. The sequence is repeated as necessary to reach the desired mixing time.

**Figure 1:**
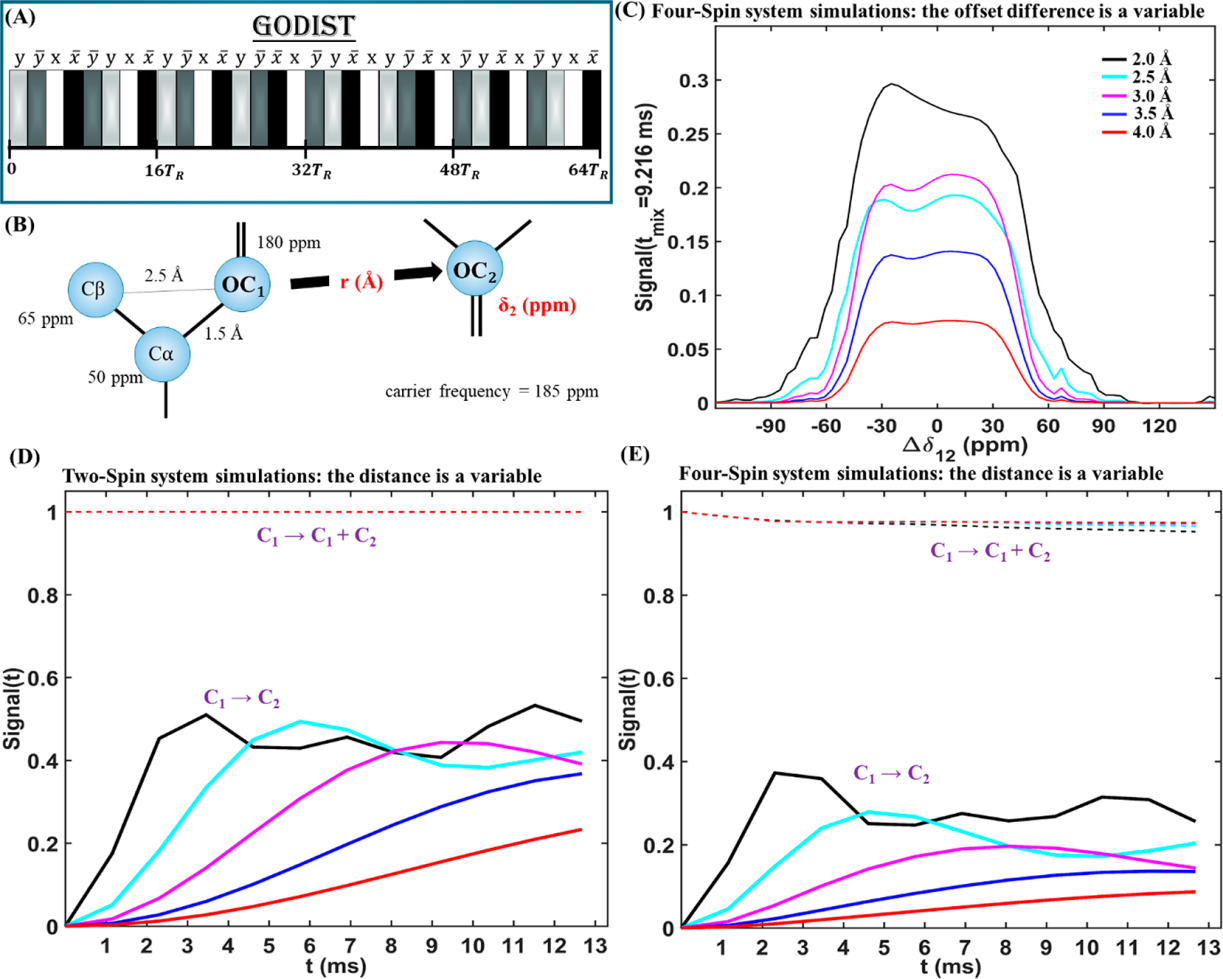
Simulations of carbonyl–carbonyl transfer using GODIST. (A) The GODIST pulse sequence element consisting of 32 2π pulses applied over 64 rotor periods (*T*_R_) with the indicated phase cycle. (B) Schematic representation of the simulated four-spin system: two carbonyl carbons (C_1_, C_2_) and two aliphatic carbons (Cα, Cβ) with chemical shift anisotropy values (respectively, in kHz): [18; 16.5; 9.75; 9]. The C_1_–C_2_ distance and the C_2_ chemical shift, δ2, were varied in the simulations of panels C–E and are shown in red. (C) Transfer as a function of the carbonyl offset difference (Δδ_12_ = 180 – δ_2_) for 2 to 4 Å distances, as indicated in the legend. (D,E) The total carbonyl signal (dashed lines) and the transferred signal (solid lines) as a function of mixing time for several distances. (D) Two-spin simulation with only carbonyl C_1_ and C_2_. (E) Four-spin simulation as shown in (B). All simulations used a 600 MHz proton Larmor frequency.

Simulations (600 MHz proton Larmor frequency) in Figure 1C were carried out on a four-spin system and show the dependence of GODIST signals on the offset differences between carbonyl spins for several distances at a mixing time of 9.216 ms. As expected, the transfer efficiency between carbonyl groups decreases with increasing distance. We observed a plateau of ±40 ppm where the transfer reaches maximal efficiency, which is broad enough to cover the carbonyl region and nearly the whole aliphatic region. On the basis of these simulations, the width of the selective transfer (the offset difference for which the transferred signal is 50% of the maximal transfer) depends only slightly on the distance between the correlated spins and equals ∼6 kHz. The transfer drops below 1% beyond a 100 ppm (15 kHz) offset difference.

Transfers are observed even in a two-spin system, thereby confirming that the sequence is a first-order zero quantum recoupling method. Simulated GODIST signals as a function of mixing time are shown in Figure 1D for the two-spin system and in Figure 1E for the four-spin system. While for the two-spin system (Figure 1D) the maximal transfer efficiency reaches ∼50%, even for longer distances, for the four-spin system (Figure 1E), the maximal transfer efficiency decreases with distance, which can be considered as an attenuated dipolar truncation effect.^39^ In both cases, however, the total signal (the sum of the remaining signal of first carbonyl spin and the signal transferred to the second carbonyl) is well-retained (dashed lines in Figure 1D,E), and the transferred signal shows only small oscillations after reaching the plateau (Figure 1E, solid lines). These properties suggest that GODIST is an ideal sequence for selectively recoupling carbonyl or aliphatic moieties and detecting weak carbon–carbon dipolar correlations.

The dependence of GODIST transfer efficiency on the experimental conditions—MAS, external magnetic field, and rf-field inhomogeneity—should be investigated. Simulations show that with increasing MAS rates, higher external magnetic fields are required for optimal GODIST performance: at 55 kHz MAS, a field of ∼600 MHz, at 83 kHz MAS, an ∼800 MHz external field, while at 111 kHz MAS, a 1200 MHz spectrometer would be ideal (Figure S1). The total initial signal is preserved robustly across various MAS frequencies and field strengths: >95% of the initial signal is retained after 9.2 ms mixing. The one exception is measurement at a 1.2 GHz spectrometer in combination with 55 kHz MAS (Figure S1C), in which case about 75% of the total signal is retained in simulation. Even in this case, undesired carbonyl-aliphatic transfers are negligible.

Figure S2A shows the simulated transferred GODIST signals as a function of mixing time with flip angle deviations up to 6% from the ideal flip angle value of 2π. These simulations show that this substantial mis-set of the rf-field power results in retention of at least 50% of the ideal transfer, suggesting that the sequence has a sufficient robustness against rf-field inhomogeneity, which is an unavoidable feature of NMR instrumentation. The transfer also has a relatively small dependence on the orientation of the chemical shift anisotropies (Figure S2B).

In order to demonstrate the selectivity of GODIST mixing, we compared it with an efficient broadband recoupling method, radio-frequency-driven recoupling (RFDR), with the carrier frequency set to either the aliphatic (Figure 2A) or the carbonyl region (Figure 2B). For proton−carbon (HC) transfers, SPECIFIC–CP conditions^69,70^ were used. For both methods, 2.304 and 25.344 ms mixing times were applied for aliphatic (A) and carbonyl (B) regions.

**Figure 2:**
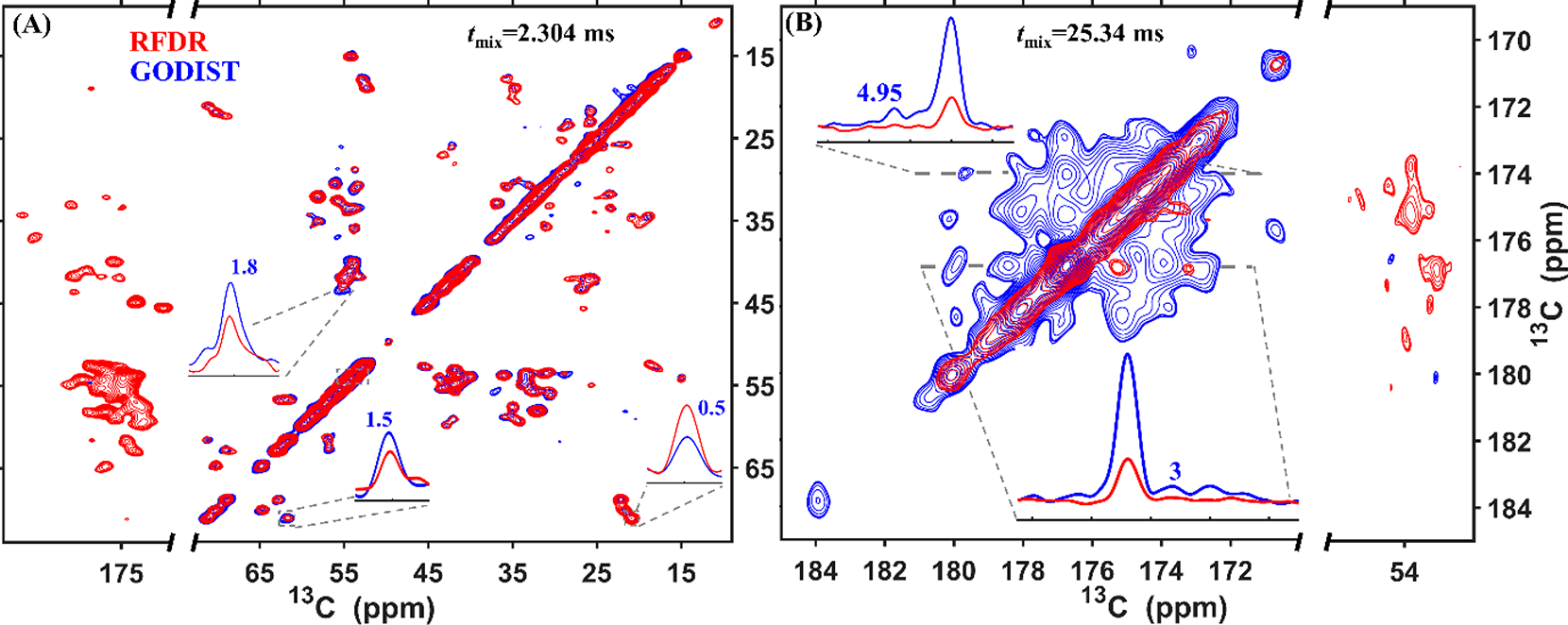
Comparison of RFDR (red) and GODIST (blue) transfers in 2D (H)CC spectra of perdeuterated microcrystalline SH3. The carbon carrier frequency was set to 50 ppm (A) and 173 ppm (B) for both sequences. For HC transfers, SPECIFIC–CP conditions^69,70^ were applied. For transfers from the aliphatic carbons (A) and from the carbonyl carbons (B), both GODIST and RFDR mixing was applied for 2.304 and 25.344 ms, respectively. For RFDR, 6 μs π pulses were applied. For GODIST, 36 μs 2π pulses were applied. All spectra were acquired at a 600 MHz spectrometer with 55.555 kHz MAS. XY-16 phase cycling^71,72^ was used for RFDR because it outperformed xy-8. Further experimental details are given in the Supporting Information (SI).

Using a sample of perdeuterated microcrystalline SH3, we found that broadband RFDR recoupling predictably mixes signals between carbonyl and aliphatic protons, while GODIST retains the signal inside the initial spectral regions, such that aliphatic–aliphatic (Figure 2A) or carbonyl–carbonyl (Figure 2B) correlations are mostly observed.

Quantification of GODIST cross-peak intensities reveals a multifold improvement in signal intensity over RFDR. Aliphatic–aliphatic correlations in GODIST are observed with a relatively modest improvement of up to 1.8-fold higher intensity (Figure 2A). The transfer efficiency of GODIST is reduced in comparison with RFDR for the largest offset differences, as occurs for threonine Cβ–Cγ correlations. The similar efficiency for both methods is explained by the fact that only carbonyl and aromatic spins lie outside the recoupling bandwidth, while the majority of carbon spins in the protein are aliphatic with strong, one-bond couplings to other aliphatic moieties.

More strikingly, Figure 2B shows a dramatic improvement in the number of observable correlations when GODIST is used for carbonyl recoupling. While carbonyl–carbonyl RFDR cross-peaks are at or below the noise level, GODIST cross-peak intensities are up to 4.95-fold higher than the noise level. At the same time, the diagonal in GODIST spectra is significantly more intense, about 3-fold, than in RFDR. This is a consequence of the carbonyl signal transfer to the aliphatic region in the case of RFDR.

Using a lipid bilayer sample of uniformly ^13^C,^15^N-labeled influenza A M2, we performed additional 2D experiments to evaluate the efficiency of GODIST for a nondeuterated sample (Figure S3). Consistent with the deuterated sample, good retention of the initial signal was observed, aliphatic–carbonyl correlations were suppressed, and in this case, an increase in intensity is observed for some aliphatic–aliphatic cross-peaks. As with SH3, the total carbonyl signal in the GODIST spectra of M2 is well preserved compared with RFDR (Figure S4).

In general, good agreement is observed between the experimental results and the simulations. Aliphatic–aliphatic transfers are not sensitive to the carrier frequency in the region between 70 and 10 ppm (Figure S5), and aliphatic–carbonyl transfers are well-suppressed. However, for carbon spins with large offsets compared with the carrier frequency (≳100 ppm), off-resonance effects^73^ decrease the efficiency of GODIST. While at a carrier frequency of 140 ppm the aromatic–aromatic correlations are readily detected (Figure S6), aliphatic–aliphatic transfers are hardly observed, and some carbonyl–aliphatic transfer occurs. Moreover, the large offset distorts the diagonal of the aliphatic region, in particular for methyl groups.

We also acquired 2D experiments with three other selective methods—MODIST,^68^ DREAM,^22,23^ and SPR5_4_ pulses^74^ (Figure S7A)—that show lower efficiency carbonyl–carbonyl correlations. Figure S7B shows GODIST efficiency at a 1200 MHz spectrometer. While GODIST’s performance deteriorated under these conditions, we still were able to detect carbonyl–carbonyl correlations up to 3.2-fold higher than the noise level.

The additional dimension provided by proton-detected 3D spectra is essential for resolving unambiguous correlations that are used for protein structure determination.^8^ We, therefore, designed 3D, proton-detected versions of ^13^C–^13^C correlation experiments. Figure 3A shows the ^13^C–^13^C projection of the (H)COCO(N)H^GODIST^ spectrum of perdeuterated microcrystalline SH3, with the assignment of selected peaks on the basis of previously determined chemical shifts.^75,76^ Most correlations belong to spins ∼3.4 Å apart; however, the long mixing time of ∼25 ms allowed the detection of 16 long-range and 3 medium-range carbonyl–carbonyl correlations up to about 5 Å (Table S1 and Figure S8), which arose due to a combination of relayed and direct transfers.

**Figure 3:**
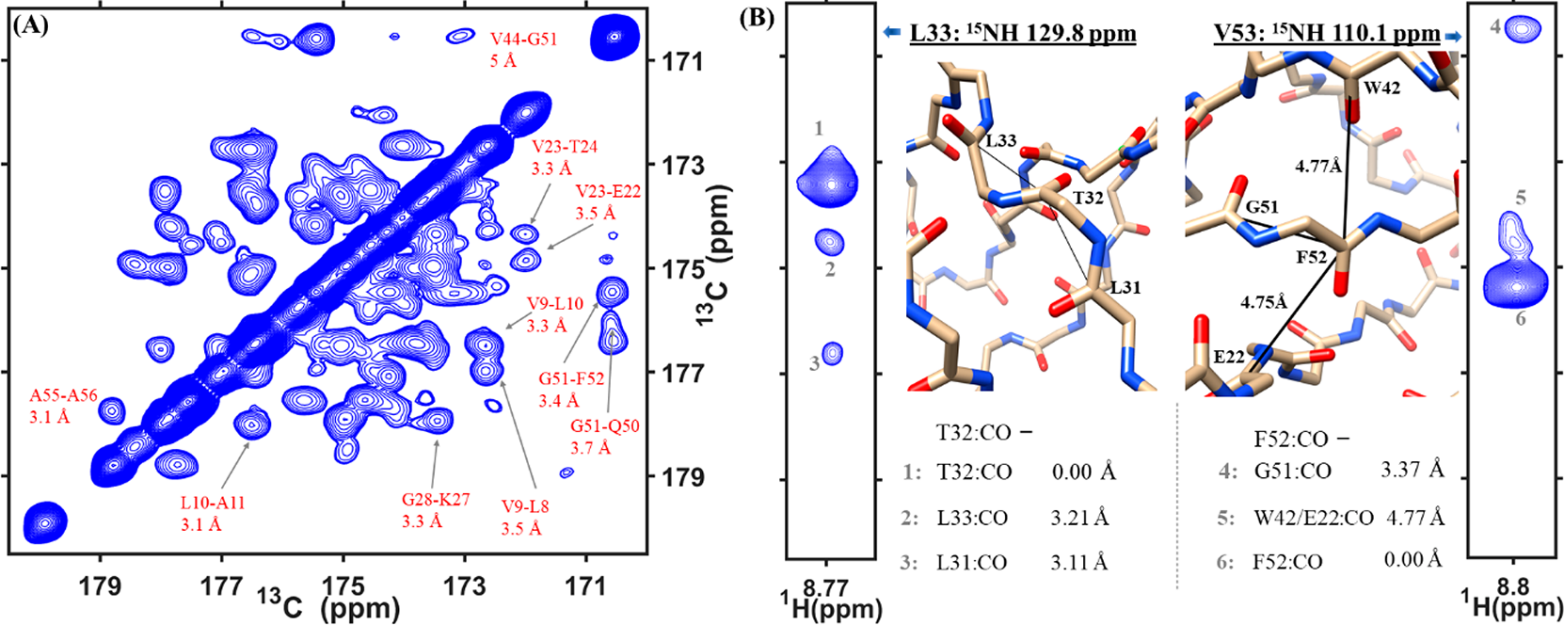
(A) ^13^C–^13^C projection of the 3D (H)COCO(N)H^GODIST^ spectrum of perdeuterated microcrystalline SH3 recorded with 25.344 ms of mixing. (B) Two strips extracted from the 3D (H)CO(CO)NH^GODIST^ spectrum at the nitrogen frequencies of L33 and V53. Distances indicated are taken from the crystal structure of SH3 (PDB code:2NUZ). Data was acquired at a 600 MHz spectrometer at 55.555 kHz MAS. The carbon carrier frequency was set to 185 ppm for the duration of mixing (further experimental details in the SI).

Figure 3B shows two strips from a 3D (H)CO(CO)NH^GODIST^ spectrum. For T32 (left), we observed two carbonyl–carbonyl cross-peaksto neighboring residues. For F52 (right), a single neighboring residue, G51, was observed, and a second cross-peak to F52 was an ambiguous correlation that can be assigned to W42 and E22, both of which are long-range correlations (4.77 and 4.75 Å in the crystal structure, PDB ID 2NUZ). The F52–V53 correlation is not present in the strip, which is likely explained by lower initial intensity at residue V53 because of the neighboring residue, P54, lacking an amide proton.

The 3D (H)CO(CO)NH^GODIST^ experiment performed similarly well for the influenza A M2 membrane protein (Figure 4). We normalized the intensities of the cross-peaks (*t*_mix_ = 9.216 ms) with peak intensities measured at 0 mixing. In each strip shown, only one correlation could be identified unambiguously, since the second one overlaps with the diagonal. On average, about 7% of the initial signal (zero mixing time) was transferred to the closest backbone carbonyl spin.

**Figure 4:**
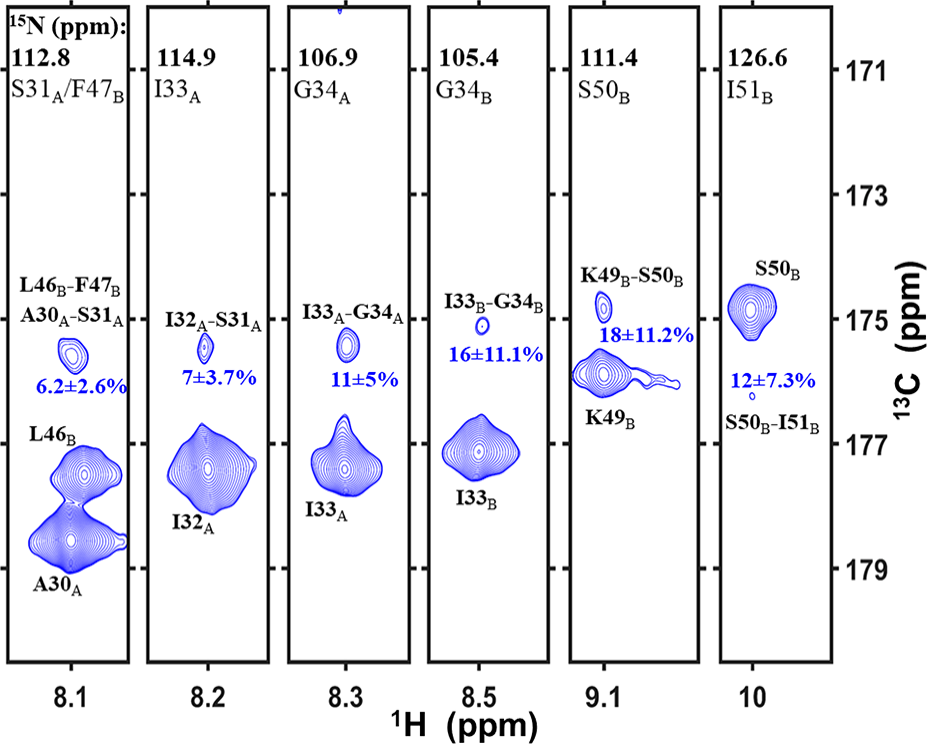
Six strips extracted from the 3D (H)CO(CO)NH^GODIST^ spectrum (9.216 ms mixing) at the amide nitrogen frequencies. The chemical shifts of nitrogen, carbon, and protons were taken from Movellan et al.^77^ The intensities of the correlated peaks are normalized with the peak intensities from the same experiment at zero mixing. Spectra were acquired at a 600 MHz spectrometer at 55.555 kHz MAS. The carbon carrier frequency was set to 185 ppm for the duration of mixing (further experimental details in the SI).

In summary, we introduced GODIST, a selective recoupling method suitable for systems with a large offset difference. This first-order recoupling sequence, designed with ^13^C resonances in mind, makes possible the detection of carbonyl–carbonyl correlations between spins up to about 5 Å in distance. The width of the selective transfer allows suppression of aliphatic–carbonyl correlations, while high retention of the initial signal allows the use of long mixing times, which is crucial for detecting longer carbon–carbon distances. We also demonstrated the efficiency and the robustness of the GODIST sequence against changes in carrier frequency position and flip angle. Comparison of GODIST and RFDR spectra showed a particular improvement for carbonyl–carbonyl cross-peaks, allowing us to identify 16 long-range correlations for SH3. We anticipate 3D (H)COCO(N)H^GODIST^ and (H)CO(CO)NH^GODIST^ experiments to facilitate protein assignment and structure determination through the detection of both sequential inter-residue carbonyl–carbonyl correlations, as well as long-range correlations.

## Supporting Information Available

Numerical simulations of GODIST, additional experimental data using GODIST and RFDR for dipolar recoupling, experimental parameters (Figures S9–S15), and Bruker Topspin pulse programs implementing the GODIST sequence (PDF)

## References

[ref1] De Paëpe G. Dipolar Recoupling in Magic Angle Spinning Solid-State Nuclear Magnetic Resonance. Annu. Rev. Phys. Chem. 2012, 63 (1), 661–684. 10.1146/annurev-physchem-032511-143726. 22404583

[ref2] Ji Y.; Liang L.; Bao X.; Hou G. Recent Progress in Dipolar Recoupling Techniques under Fast MAS in Solid-State NMR Spectroscopy. Solid State Nucl. Magn. Reson. 2021, 112, 101711 10.1016/j.ssnmr.2020.101711. 33508579

[ref3] Liang L.; Ji Y.; Chen K.; Gao P.; Zhao Z.; Hou G. Solid-State NMR Dipolar and Chemical Shift Anisotropy Recoupling Techniques for Structural and Dynamical Studies in Biological Systems. Chem. Rev. 2022, 122 (10), 9880–9942. 10.1021/acs.chemrev.1c00779. 35006680

[ref4] Nishiyama Y.; Hou G.; Agarwal V.; Su Y.; Ramamoorthy A. Ultrafast Magic Angle Spinning Solid-State NMR Spectroscopy: Advances in Methodology and Applications. Chem. Rev. 2023, 123, 918 10.1021/acs.chemrev.2c00197. 36542732 PMC10319395

[ref5] Zhou D. H.; Shah G.; Cormos M.; Mullen C.; Sandoz D.; Rienstra C. M. Proton-Detected Solid-State NMR Spectroscopy of Fully Protonated Proteins at 40 kHz Magic-Angle Spinning. J. Am. Chem. Soc. 2007, 129 (38), 11791–11801. 10.1021/ja073462m. 17725352

[ref6] Zhou D. H.; Nieuwkoop A. J.; Berthold D. A.; Comellas G.; Sperling L. J.; Tang M.; Shah G. J.; Brea E. J.; Lemkau L. R.; Rienstra C. M. Solid-State NMR Analysis of Membrane Proteins and Protein Aggregates by Proton Detected Spectroscopy. J. Biomol. NMR 2012, 54 (3), 291–305. 10.1007/s10858-012-9672-z. 22986689 PMC3484199

[ref7] Andreas L. B.; Le Marchand T.; Jaudzems K.; Pintacuda G. High-Resolution Proton-Detected NMR of Proteins at Very Fast MAS. J. Magn. Reson. 2015, 253, 36–49. 10.1016/j.jmr.2015.01.003. 25797003

[ref8] Le Marchand T.; Schubeis T.; Bonaccorsi M.; Paluch P.; Lalli D.; Pell A. J.; Andreas L. B.; Jaudzems K.; Stanek J.; Pintacuda G. 1H-Detected Biomolecular NMR under Fast Magic-Angle Spinning. Chem. Rev. 2022, 122 (10), 9943–10018. 10.1021/acs.chemrev.1c00918. 35536915 PMC9136936

[ref9] Reif B. Deuteration for High-Resolution Detection of Protons in Protein Magic Angle Spinning (MAS) Solid-State NMR. Chem. Rev. 2022, 122 (10), 10019–10035. 10.1021/acs.chemrev.1c00681. 34870415

[ref10] Reif B.; Ashbrook S. E.; Emsley L.; Hong M. Solid-State NMR Spectroscopy. Nat. Rev. Methods Primer 2021, 1 (1), 1–23. 10.1038/s43586-020-00002-1. PMC8341432 34368784

[ref11] Ahlawat S.; Mote K. R.; Lakomek N.-A.; Agarwal V. Solid-State NMR: Methods for Biological Solids. Chem. Rev. 2022, 122 (10), 9643–9737. 10.1021/acs.chemrev.1c00852. 35238547

[ref12] Mandala V. S.; Williams J. K.; Hong M. Structure and Dynamics of Membrane Proteins from Solid-State NMR. Annu. Rev. Biophys. 2018, 47, 201–222. 10.1146/annurev-biophys-070816-033712. 29498890 PMC6312106

[ref13] van der Wel P. C. A. Insights into Protein Misfolding and Aggregation Enabled by Solid-State NMR Spectroscopy. Solid State Nucl. Magn. Reson. 2017, 88, 1–14. 10.1016/j.ssnmr.2017.10.001. 29035839 PMC5705391

[ref14] Quinn C. M.; Polenova T. Structural Biology of Supramolecular Assemblies by Magic-Angle Spinning NMR Spectroscopy. Q. Rev. Biophys. 2017, 50, e1 10.1017/S0033583516000159. 28093096 PMC5483179

[ref15] Porat-Dahlerbruch G.; Goldbourt A.; Polenova T. Virus Structures and Dynamics by Magic-Angle Spinning NMR. Annu. Rev. Virol. 2021, 8 (1), 219–237. 10.1146/annurev-virology-011921-064653. 34586870 PMC8973440

[ref16] Yan S.; Suiter C. L.; Hou G.; Zhang H.; Polenova T. Probing Structure and Dynamics of Protein Assemblies by Magic Angle Spinning NMR Spectroscopy. Acc. Chem. Res. 2013, 46 (9), 2047–2058. 10.1021/ar300309s. 23402263 PMC3748245

[ref17] Tycko R. Molecular Structure of Aggregated Amyloid-β: Insights from Solid-State Nuclear Magnetic Resonance. Cold Spring Harb. Perspect. Med. 2016, 6 (8), a024083 10.1101/cshperspect.a024083. 27481836 PMC4968170

[ref18] Tycko R. Solid-State NMR Studies of Amyloid Fibril Structure. Annu. Rev. Phys. Chem. 2011, 62 (1), 279–299. 10.1146/annurev-physchem-032210-103539. 21219138 PMC3191906

[ref19] Ladizhansky V. Nuclear Magnetic Resonance Spectroscopy | Solid-State NMR of Macromolecules. Encyclopedia of Analytical Science 2018, 414–426. 10.1016/B978-0-12-409547-2.14083-1.

[ref20] Bennett A. E.; Griffin R. G.; Ok J. H.; Vega S. Chemical Shift Correlation Spectroscopy in Rotating Solids: Radio Frequency-driven Dipolar Recoupling and Longitudinal Exchange. J. Chem. Phys. 1992, 96 (11), 8624–8627. 10.1063/1.462267.

[ref21] Ishii Y. 13C-13C Dipolar Recoupling under Very Fast Magic Angle Spinning in Solid-State Nuclear Magnetic Resonance: Applications to Distance Measurements, Spectral Assignments, and High-Throughput Secondary-Structure Determination. J. Chem. Phys. 2001, 114 (19), 8473–8483. 10.1063/1.1359445.

[ref22] Verel R.; Baldus M.; Ernst M.; Meier B. H. A Homonuclear Spin-Pair Filter for Solid-State NMR Based on Adiabatic-Passage Techniques. Chem. Phys. Lett. 1998, 287 (3), 421–428. 10.1016/S0009-2614(98)00172-9.

[ref23] Verel R.; Ernst M.; Meier B. H. Adiabatic Dipolar Recoupling in Solid-State NMR: The DREAM Scheme. J. Magn. Reson. 2001, 150 (1), 81–99. 10.1006/jmre.2001.2310. 11330986

[ref24] Manolikas T.; Herrmann T.; Meier B. H. Protein Structure Determination from 13C Spin-Diffusion Solid-State NMR Spectroscopy. J. Am. Chem. Soc. 2008, 130 (12), 3959–3966. 10.1021/ja078039s. 18321098

[ref25] Teymoori G.; Pahari B.; Edén M. Low-Power Broadband Homonuclear Dipolar Recoupling in MAS NMR by Two-Fold Symmetry Pulse Schemes for Magnetization Transfers and Double-Quantum Excitation. J. Magn. Reson. 2015, 261, 205–220. 10.1016/j.jmr.2015.09.004. 26515279

[ref26] Carravetta M.; Edén M.; Zhao X.; Brinkmann A.; Levitt M. H. Symmetry Principles for the Design of Radiofrequency Pulse Sequences in the Nuclear Magnetic Resonance of Rotating Solids. Chem. Phys. Lett. 2000, 321 (3), 205–215. 10.1016/S0009-2614(00)00340-7.

[ref27] Brinkmann A.; Schmedt auf der Günne J.; Levitt M. H. Homonuclear Zero-Quantum Recoupling in Fast Magic-Angle Spinning Nuclear Magnetic Resonance. J. Magn. Reson. 2002, 156 (1), 79–96. 10.1006/jmre.2002.2525. 12081445

[ref28] Hohwy M.; Rienstra C. M.; Jaroniec C. P.; Griffin R. G. Fivefold Symmetric Homonuclear Dipolar Recoupling in Rotating Solids: Application to Double Quantum Spectroscopy. J. Chem. Phys. 1999, 110 (16), 7983–7992. 10.1063/1.478702.

[ref29] Nielsen A. B.; Jain S. K.; Nielsen N. Chr. Low-Power Homonuclear Dipolar Recoupling Using Supercycled Symmetry-Based and Exponentially-Modulated Pulse Sequences. Chem. Phys. Lett. 2011, 503 (4-6), 310–315. 10.1016/j.cplett.2010.12.084.

[ref30] Rienstra C. M.; Hatcher M. E.; Mueller L. J.; Sun; Fesik S. W.; Griffin R. G. Efficient Multispin Homonuclear Double-Quantum Recoupling for Magic-Angle Spinning NMR: 13C-13C Correlation Spectroscopy of U-13C-Erythromycin A. J. Am. Chem. Soc. 1998, 120 (41), 10602–10612. 10.1021/ja9810181.

[ref31] Kristiansen P. E.; Mitchell D. J.; Evans J. N. S. Double-Quantum Dipolar Recoupling at High Magic-Angle Spinning Rates. J. Magn. Reson. 2002, 157 (2), 253–266. 10.1006/jmre.2002.2594. 12323144

[ref32] Higman V. A. Solid-State MAS NMR Resonance Assignment Methods for Proteins. Prog. Nucl. Magn. Reson. Spectrosc. 2018, 106–107, 37–65. 10.1016/j.pnmrs.2018.04.002. 31047601

[ref33] Kupče E.; Mote K. R.; Webb A.; Madhu P. K.; Claridge T. D. W. Multiplexing Experiments in NMR and Multi-Nuclear MRI. Prog. Nucl. Magn. Reson. Spectrosc. 2021, 124–125, 1–56. 10.1016/j.pnmrs.2021.03.001. 34479710

[ref34] Aguion P. I.; Marchanka A. Strategies for RNA Resonance Assignment by 13C/15N- and 1H-Detected Solid-State NMR Spectroscopy. Front. Mol. Biosci. 2021, 8, 743181 10.3389/fmolb.2021.743181. 34746232 PMC8563574

[ref35] Sun B.-Q.; Costa P. R.; Kocisko D.; Lansbury P. T.; Griffin R. G. Internuclear Distance Measurements in Solid State Nuclear Magnetic Resonance: Dipolar Recoupling via Rotor Synchronized Spin Locking. J. Chem. Phys. 1995, 102 (2), 702–707. 10.1063/1.469182.

[ref36] Ladizhansky V. Homonuclear Dipolar Recoupling Techniques for Structure Determination in Uniformly 13C-Labeled Proteins. Solid State Nucl. Magn. Reson. 2009, 36 (3), 119–128. 10.1016/j.ssnmr.2009.07.003. 19729285

[ref37] Saalwächter K. Robust NMR Approaches for the Determination of Homonuclear Dipole-Dipole Coupling Constants in Studies of Solid Materials and Biomolecules. ChemPhysChem 2013, 14 (13), 3000–3014. 10.1002/cphc.201300254. 23754803

[ref38] Tycko R.; Dabbagh G. Measurement of Nuclear Magnetic Dipole—Dipole Couplings in Magic Angle Spinning NMR. Chem. Phys. Lett. 1990, 173 (5), 461–465. 10.1016/0009-2614(90)87235-J.

[ref39] Bayro M. J.; Huber M.; Ramachandran R.; Davenport T. C.; Meier B. H.; Ernst M.; Griffin R. G. Dipolar Truncation in Magic-Angle Spinning NMR Recoupling Experiments. J. Chem. Phys. 2009, 130 (11), 114506 10.1063/1.3089370. 19317544 PMC4435003

[ref40] Mithu V. S.; Bakthavatsalam S.; Madhu P. K. 13C-13C Homonuclear Recoupling in Solid-State Nuclear Magnetic Resonance at a Moderately High Magic-Angle-Spinning Frequency. PLoS One 2013, 8 (1), e50504 10.1371/journal.pone.0050504. 23326308 PMC3542364

[ref41] Takegoshi K.; Nakamura S.; Terao T. 13C-1H Dipolar-Assisted Rotational Resonance in Magic-Angle Spinning NMR. Chem. Phys. Lett. 2001, 344 (5), 631–637. 10.1016/S0009-2614(01)00791-6.

[ref42] Szeverenyi N. M.; Sullivan M. J.; Maciel G. E. Observation of Spin Exchange by Two-Dimensional Fourier Transform 13C Cross Polarization-Magic-Angle Spinning. J. Magn. Reson. 1969 1982, 47 (3), 462–475. 10.1016/0022-2364(82)90213-X.

[ref43] Scholz I.; Huber M.; Manolikas T.; Meier B. H.; Ernst M. MIRROR Recoupling and Its Application to Spin Diffusion under Fast Magic-Angle Spinning. Chem. Phys. Lett. 2008, 460 (1), 278–283. 10.1016/j.cplett.2008.05.058.

[ref44] Morcombe C. R.; Gaponenko V.; Byrd R. A.; Zilm K. W. Diluting Abundant Spins by Isotope Edited Radio Frequency Field Assisted Diffusion. J. Am. Chem. Soc. 2004, 126 (23), 7196–7197. 10.1021/ja047919t. 15186155

[ref45] Weingarth M.; Bodenhausen G.; Tekely P. Broadband Carbon-13 Correlation Spectra of Microcrystalline Proteins in Very High Magnetic Fields. J. Am. Chem. Soc. 2009, 131 (39), 13937–13939. 10.1021/ja9036143. 19743847

[ref46] Hu B.; Lafon O.; Trébosc J.; Chen Q.; Amoureux J.-P. Broad-Band Homo-Nuclear Correlations Assisted by 1H Irradiation for Bio-Molecules in Very High Magnetic Field at Fast and Ultra-Fast MAS Frequencies. J. Magn. Reson. 2011, 212 (2), 320–329. 10.1016/j.jmr.2011.07.011. 21873091

[ref47] Hou G.; Yan S.; Sun S.; Han Y.; Byeon I.-J. L.; Ahn J.; Concel J.; Samoson A.; Gronenborn A. M.; Polenova T. Spin Diffusion Driven by R-Symmetry Sequences: Applications to Homonuclear Correlation Spectroscopy in MAS NMR of Biological and Organic Solids. J. Am. Chem. Soc. 2011, 133 (11), 3943–3953. 10.1021/ja108650x. 21361320 PMC3148607

[ref48] De Paëpe G.; Lewandowski J. R.; Loquet A.; Böckmann A.; Griffin R. G. Proton Assisted Recoupling and Protein Structure Determination. J. Chem. Phys. 2008, 129 (24), 245101 10.1063/1.3036928. 19123534 PMC2755343

[ref49] Lewandowski J.; De Paëpe G.; Griffin R. G. Proton Assisted Insensitive Nuclei Cross Polarization. J. Am. Chem. Soc. 2007, 129 (4), 728–729. 10.1021/ja0650394. 17243786 PMC2518536

[ref50] Demers J.-P.; Fricke P.; Shi C.; Chevelkov V.; Lange A. Structure Determination of Supra-Molecular Assemblies by Solid-State NMR: Practical Considerations. Prog. Nucl. Magn. Reson. Spectrosc. 2018, 109, 51–78. 10.1016/j.pnmrs.2018.06.002. 30527136

[ref51] Verardi R.; Traaseth N. J.; Masterson L. R.; Vostrikov V. V.; Veglia G. Isotope Labeling for Solution and Solid-State NMR Spectroscopy of Membrane Proteins. Adv. Exp. Med. Biol. 2012, 992, 35–62. 10.1007/978-94-007-4954-2_3. 23076578 PMC3555569

[ref52] Eddy M. T.; Belenky M.; Sivertsen A.; Griffin R. G.; Herzfeld J. Selectively Dispersed Isotope Labeling for Protein Structure Determination by Magic Angle Spinning NMR. J. Biomol. NMR 2013, 57 (2), 129–139. 10.1007/s10858-013-9773-3. 23990199 PMC3793012

[ref53] Fasshuber H. K.; Demers J.-P.; Chevelkov V.; Giller K.; Becker S.; Lange A. Specific 13C Labeling of Leucine, Valine and Isoleucine Methyl Groups for Unambiguous Detection of Long-Range Restraints in Protein Solid-State NMR Studies. J. Magn. Reson. 2015, 252, 10–19. 10.1016/j.jmr.2014.12.013. 25625825

[ref54] Lacabanne D.; Meier B. H.; Böckmann A. Selective Labeling and Unlabeling Strategies in Protein Solid-State NMR Spectroscopy. J. Biomol. NMR 2018, 71 (3), 141–150. 10.1007/s10858-017-0156-z. 29197975

[ref55] Bayro M. J.; Maly T.; Birkett N. R.; Dobson C. M.; Griffin R. G. Long-Range Correlations between Aliphatic 13C Nuclei in Protein MAS NMR Spectroscopy. Angew. Chem., Int. Ed. 2009, 48 (31), 5708–5710. 10.1002/anie.200901520. PMC2982996 19562810

[ref56] Chevelkov V.; Giller K.; Becker S.; Lange A. Efficient CO-CA Transfer in Highly Deuterated Proteins by Band-Selective Homonuclear Cross-Polarization. J. Magn. Reson. 2013, 230, 205–211. 10.1016/j.jmr.2013.02.021. 23558259

[ref57] Westfeld T.; Verel R.; Ernst M.; Böckmann A.; Meier B. H. Properties of the DREAM Scheme and Its Optimization for Application to Proteins. J. Biomol. NMR 2012, 53 (2), 103–112. 10.1007/s10858-012-9627-4. 22562365

[ref58] Hu K.-N.; Tycko R. Zero-Quantum Frequency-Selective Recoupling of Homonuclear Dipole-Dipole Interactions in Solid State Nuclear Magnetic Resonance. J. Chem. Phys. 2009, 131 (4), 045101 10.1063/1.3176874. 19655922 PMC2728366

[ref59] Hu K.-N.; Qiang W.; Bermejo G. A.; Schwieters C. D.; Tycko R. Restraints on Backbone Conformations in Solid State NMR Studies of Uniformly Labeled Proteins from Quantitative Amide 15N-15N and Carbonyl 13C-13C Dipolar Recoupling Data. J. Magn. Reson. 2012, 218, 115–127. 10.1016/j.jmr.2012.03.001. 22449573 PMC3568759

[ref60] Paravastu A. K.; Tycko R. Frequency-Selective Homonuclear Dipolar Recoupling in Solid State NMR. J. Chem. Phys. 2006, 124 (19), 194303 10.1063/1.2192516. 16729810 PMC1851697

[ref61] Hohwy M.; Rienstra C. M.; Griffin R. G. Band-Selective Homonuclear Dipolar Recoupling in Rotating Solids. J. Chem. Phys. 2002, 117 (10), 4973–4987. 10.1063/1.1488136.

[ref62] Matsuki Y.; Akutsu H.; Fujiwara T. Band-Selective Recoupling of Homonuclear Double-Quantum Dipolar Interaction with a Generalized Composite 0° Pulse: Application to 13C Aliphatic Region-Selective Magnetization Transfer in Solids. J. Magn. Reson. 2003, 162 (1), 54–66. 10.1016/S1090-7807(02)00191-X. 12762983

[ref63] Xiao H.; Zhang Z.; Zhao Y.; Yang J. Spectral Editing of Alanine, Serine, and Threonine in Uniformly Labeled Proteins Based on Frequency-Selective Homonuclear Recoupling in Solid-State NMR. J. Biomol. NMR 2021, 75 (4), 193–202. 10.1007/s10858-021-00367-9. 33890210

[ref64] Xiao H.; Zhang Z.; Yang J. Theory of Frequency-Selective Homonuclear Dipolar Recoupling in Solid-State NMR. J. Chem. Phys. 2021, 155, 174105 10.1063/5.0065396. 34742189

[ref65] Zhang Z.; Liu H.; Deng J.; Tycko R.; Yang J. Optimization of Band-Selective Homonuclear Dipolar Recoupling in Solid-State NMR by a Numerical Phase Search. J. Chem. Phys. 2019, 150 (15), 154201 10.1063/1.5092986. 31005077 PMC6474779

[ref66] Kehlet C.; Nielsen J. T.; Tosner Z.; Nielsen N. Chr. Resolution-Enhanced Solid-State NMR 13C-13C Correlation Spectroscopy by Optimal Control Dipolar-Driven Spin-State-Selective Coherence Transfer. J. Phys. Chem. Lett. 2011, 2 (6), 543–547. 10.1021/jz101695d.

[ref78] Chavez M.; Ernst M. Interaction Frames in Solid-State NMR: A Case Study for Chemical-Shift-Selective Irradiation Schemes. Solid State Nucl. Magn. Reson. 2022, 122, 101834 10.1016/j.ssnmr.2022.101834. 36327552

[ref67] Potnuru L. R.; Duong N. T.; Ahlawat S.; Raran-Kurussi S.; Ernst M.; Nishiyama Y.; Agarwal V. Accuracy of 1H-1H Distances Measured Using Frequency Selective Recoupling and Fast Magic-Angle Spinning. J. Chem. Phys. 2020, 153 (8), 084202 10.1063/5.0019717. 32872876

[ref68] Nimerovsky E.; Najbauer E. E.; Movellan K. T.; Xue K.; Becker S.; Andreas L. B. Modest Offset Difference Internuclear Selective Transfer via Homonuclear Dipolar Coupling. J. Phys. Chem. Lett. 2022, 13 (6), 1540–1546. 10.1021/acs.jpclett.1c03871. 35133845 PMC8859849

[ref69] Baldus M.; Petkova A. T.; Herzfeld J.; Griffin R. G. Cross Polarization in the Tilted Frame: Assignment and Spectral Simplification in Heteronuclear Spin Systems. Mol. Phys. 1998, 95 (6), 1197–1207. 10.1080/00268979809483251.

[ref70] Laage S.; Marchetti A.; Sein J.; Pierattelli R.; Sass H. J.; Grzesiek S.; Lesage A.; Pintacuda G.; Emsley L. Band-Selective 1H-13C Cross-Polarization in Fast Magic Angle Spinning Solid-State NMR Spectroscopy. J. Am. Chem. Soc. 2008, 130 (51), 17216–17217. 10.1021/ja805926d. 19053413

[ref71] Gullion T.; Baker D. B.; Conradi M. S. New, Compensated Carr-Purcell Sequences. J. Magn. Reson. 1969 1990, 89 (3), 479–484. 10.1016/0022-2364(90)90331-3.

[ref72] Zhang R.; Nishiyama Y.; Sun P.; Ramamoorthy A. Phase Cycling Schemes for Finite-Pulse-RFDR MAS Solid State NMR Experiments. J. Magn. Reson. 2015, 252, 55–66. 10.1016/j.jmr.2014.12.010. 25655451 PMC4380770

[ref73] Abragam A. The Principles of Nuclear Magnetism; International Series of Monographs on Physics, Vol. 32; Clarendon Press: Oxford, UK, 1989.

[ref74] Zhang Z.; Oss A.; Org M.-L.; Samoson A.; Li M.; Tan H.; Su Y.; Yang J. Selectively Enhanced 1H-1H Correlations in Proton-Detected Solid-State NMR under Ultrafast MAS Conditions. J. Phys. Chem. Lett. 2020, 11 (19), 8077–8083. 10.1021/acs.jpclett.0c02412. 32880459

[ref75] Pauli J.; Baldus M.; van Rossum B.; de Groot H.; Oschkinat H. Backbone and Side-Chain 13C and 15N Signal Assignments of the α-Spectrin SH3 Domain by Magic Angle Spinning Solid-State NMR at 17.6 T. ChemBioChem. 2001, 2 (4), 272–281. 10.1002/1439-7633(20010401)2:4<272::AID-CBIC272>3.0.CO;2-2. 11828455

[ref76] van Rossum B.-J.; Castellani F.; Pauli J.; Rehbein K.; Hollander J.; de Groot H. J. M.; Oschkinat H. Assignment of Amide Proton Signals by Combined Evaluation of HN, NN and HNCA MAS-NMR Correlation Spectra. J. Biomol. NMR 2003, 25 (3), 217–223. 10.1023/A:1022819921584. 12652133

[ref77] Movellan K. T.; Westroth M.; Overkamp K.; Leonov A.; Becker S.; Andreas L. B. Real-time tracking of drug binding to Influenza A M2 reveals a high energy barrier. bioRxiv, April 7, 2023, 536045. 10.1101/2023.04.07.536045. PMC10285276 37363040

