# Supplementary material for: Great Offset Difference Internuclear Selective Transfer
Source: J Phys Chem Lett. 2023 Apr 20;14(16):3939–45. doi: 10.1021/acs.jpclett.3c00194 (PMC10150390; doi:10.1021/acs.jpclett.3c00194)
Supplement: Supplementary file 1 — jz3c00194_si_001.pdf [file jz3c00194_si_001.pdf]

## Supplementary Information

# Great Offset Difference Internuclear Selective Transfer

*Authors: Evgeny Nimerovsky*, Eszter Éva Najbauer, Stefan Becker & Loren B. Andreas**

Department of NMR based Structural Biology, Max Planck Institute for Multidisciplinary Sciences, Am Fassberg 11, Göttingen, Germany

***Corresponding authors**: land@nmr.mpibpc.mpg.de ORCID: 0000-0003-3216-9065 and evni@nmr.mpibpc.mpg.de ORCID: 0000-0003-3002-0718.

# Contents


SIMULATIONS .......... 2

EXPERIMENTAL DATA .......... 4

EXPERIMENTAL METHODS .......... 8

Simulations .......... 8

Sample Preparation .......... 8

Solid state NMR spectroscopy .......... 9

BRUKER PULSE PROGRAMS .......... 14

REFERENCE .......... 29


# SIMULATIONS

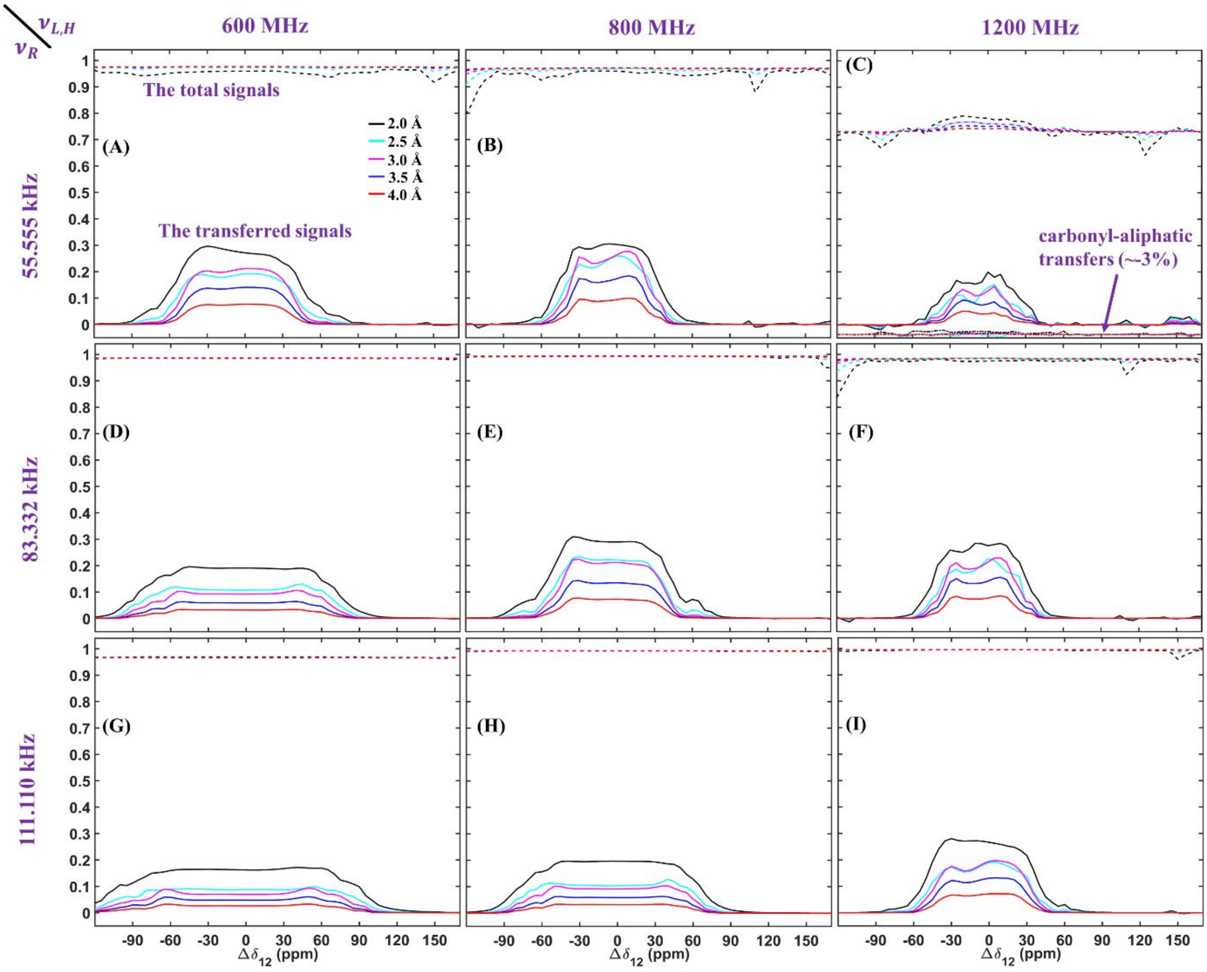


**Figure S1** Simulated transferred (solid lines) and the total carbonyl (the remaining + the transferred signal, dashed lines) GODIST signals at 9.216 ms mixing as a function of the carbonyl offset difference (between $OC_1$ and $OC_2$, $\Delta\delta_{12}$=180-$\delta_2$) for different MAS rates (rows), external magnetic fields (columns) and distances: 2.0 Å

– black lines; 2.5 Å – cyan lines; 3.0 Å – magenda lines; 3.5 Å – blue lines and 4.0 Å – red lines), as labeled. The four-spin system of Figure 1B in the main text was used with the following values for [isotropic chemical shift; chemical shift anysotropy] in ppm: the initial carbonyl spin ($OC_1$) – [180; 120]; the measured carbonyl spin ($OC_2$) – [variable; 110]; the first aliphatic carbon ($C_{\alpha 3}$) – [50;65]; the second aliphatic carbon ($C_{\beta 4}$) – [65; 60]. The carrier frequency position was at 185 ppm. In (C) the carbonyl-aliphatic ($OC_1$-$C_{\alpha 3}$) tranfers are shown as dotted lines (~3 %), while in other cases these transfers are ~0.5%.

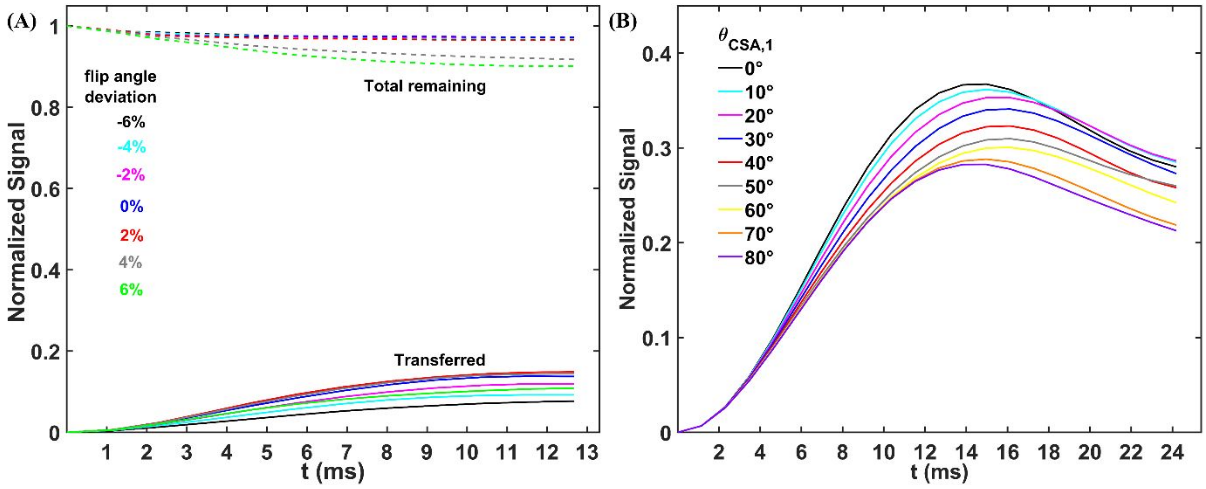


**Figure S2** The influence of RF-field inhomogeneity (A, four spin system) and relative orientation between chemical shift anisotropy (csa) tensors (B, two spin system). (A) Simulated transferred polarization (solid lines) and the total remaining polarization (signal on z + transferred signal on z, dashed lines) is shown for GODIST as a function of mixing time for different flip angle deviations: -6% - black lines; -4% - cyan lines; -2% - magenda lines; 0% (2π flip angle) – blue lines; 2% - red lines; 4% - gray lines; 6% - green lines. The four spin system (Figure 1B in the main text) was used with the following values for [isotropic chemical shift; chemical shift anysotropy] in ppm: the initial carbonyl spin ($OC_1$) – [170; 120]; the measured carbonyl spin ($OC_2$) – [176; 110]; the first aliphatic carbon ($C_{\alpha 3}$) – [50;65]; the second aliphatic carbon ($C_{\beta 4}$) – [65; 60]. The distance between $OC_1$ and $OC_2$ was 3.5 Å. (B) Simulated transferred polarization as a function of mixing time with different orientation of chemical shift anisotropy of the initial spin ($\theta_{CSA,1}$ – var) compared to the measured spin ($\theta_{CSA,2}$=0°). Only one relative angle was varied, while other two were fixed and equaled to 0°. The two spin system (Figure 1B in the main text) was used with the following values for [isotropic chemical shift (in ppm); chemical shift anysotropy (in ppm), eta[1]]: the initial carbonyl spin ($OC_1$) – [170; 120, 0.8]; the measured carbonyl spin ($OC_2$) – [176; 110, 0.8]. The distance between $OC_1$ and $OC_2$ was 3.5 Å. The simulation was

performed for 55.555 kHz MAS and 600 MHz proton Larmor frequency. The carrier frequency position was at 185 ppm.

# EXPERIMENTAL DATA

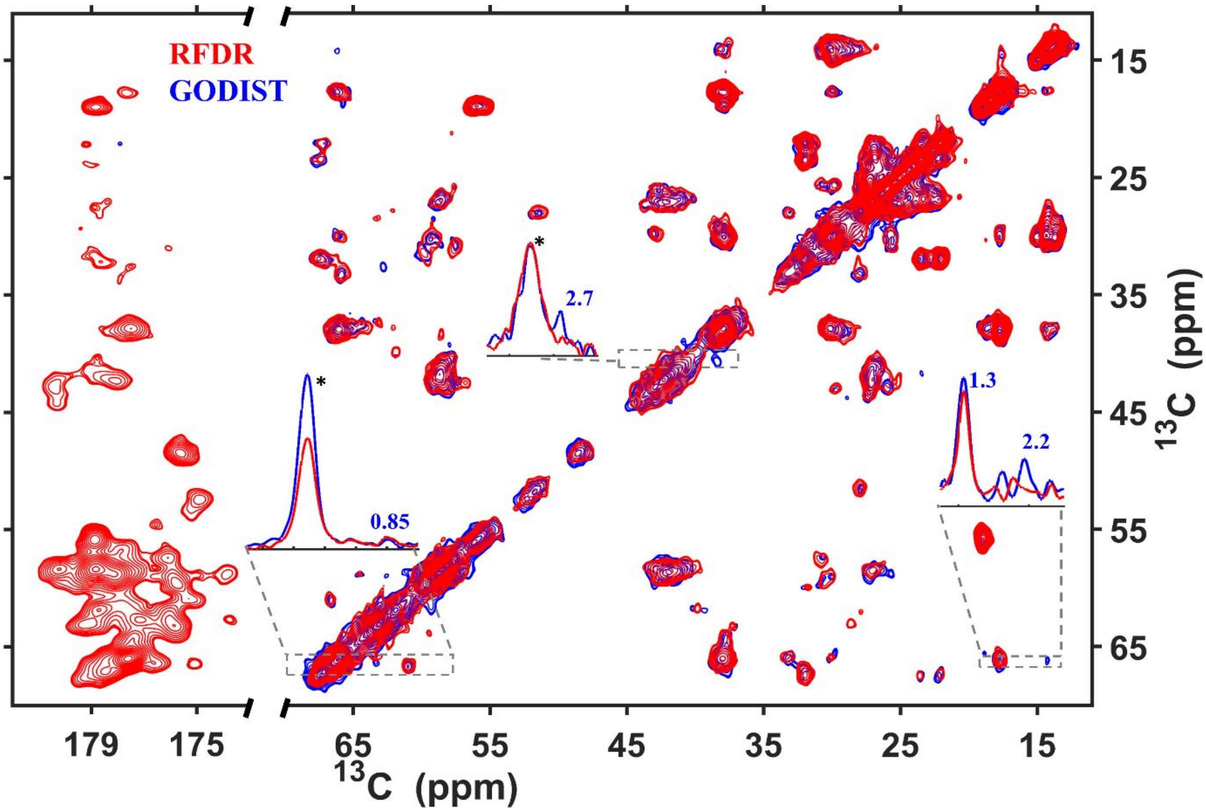


**Figure S3** Comparison of RFDR (red) and GODIST (blue) transfers in 2D (H)CC spectra of Influenza A M2. During mixing blocks (2.304 ms mixing) the carbon carrier frequency was set to 35 ppm. For RFDR 6 µs (83.33 kHz rf-field strength) π-pulses were applied. For GODIST, 36 µs (27.777 kHz rf-field strength) 2π-pulses were applied. Balck stars ‘ * ’ indicate diagonal peaks. Data were acquired at a 600 MHz spectrometer with 55.555 kHz MAS. XY16 phase cycling was used for RFDR. Full experimental details are given below in the experimental methods section.

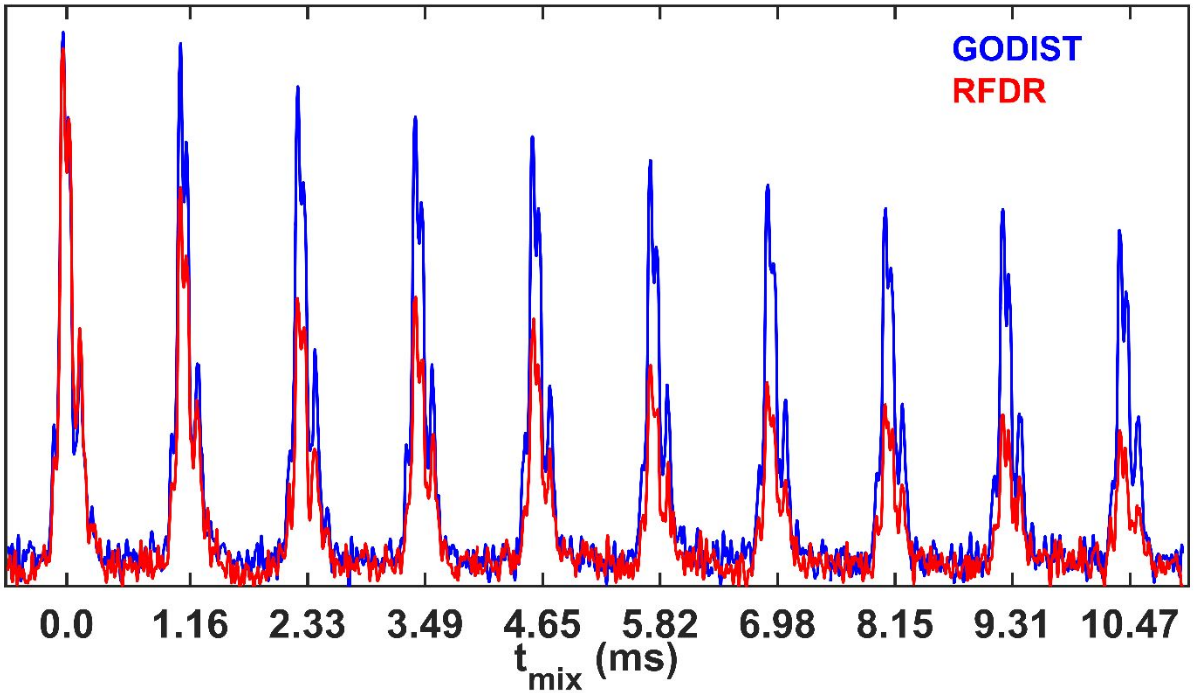


**Figure S4** Carbonyl signal as a function of mixing time for GODIST (blue) and RFDR (red) in 1D (HC)C spectra of Influenza A M2. For RFDR, 6 µs (83.333 kHz rf-field strength) π-pulses are applied and XY-16 phase cycling[2]. For GODIST, 36 µs (27.777 kHz rf-field strength) 2π-pulses were applied. The carbon carrier frequency was set to 174 ppm. Data was acquired at a 600 MHz spectrometer with 55.555 kHz MAS.

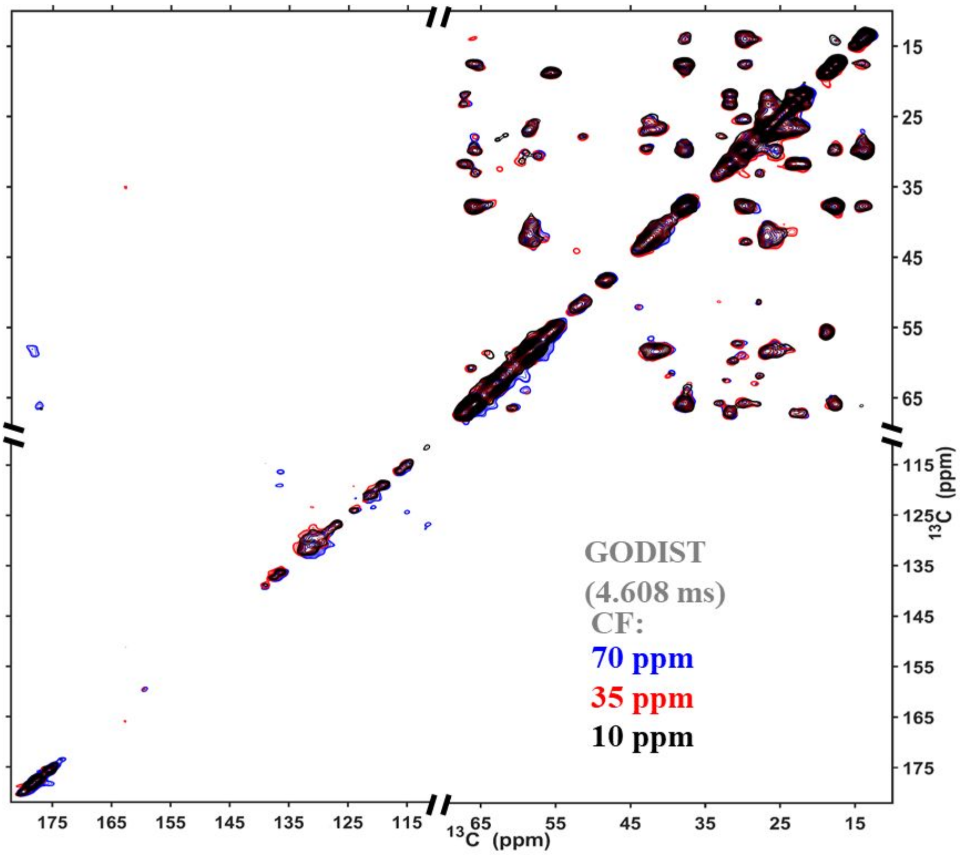

**Figure S5** 2D (H)CC$^{GODIST}$ spectra at 4.608 ms mixing of Influenza A M2 for different carrier frequency (CF) positions during the GODIST mixing: 70 ppm – blue; 35 ppm – red and 10 ppm –black. The experiments were acquired at a 600 MHz spectrometer with 55.555 kHz MAS. 36 μs (27.777 kHz rf-field strength) 2π-pulses were applied. Further experimental details are provided in the experimental methods, below.

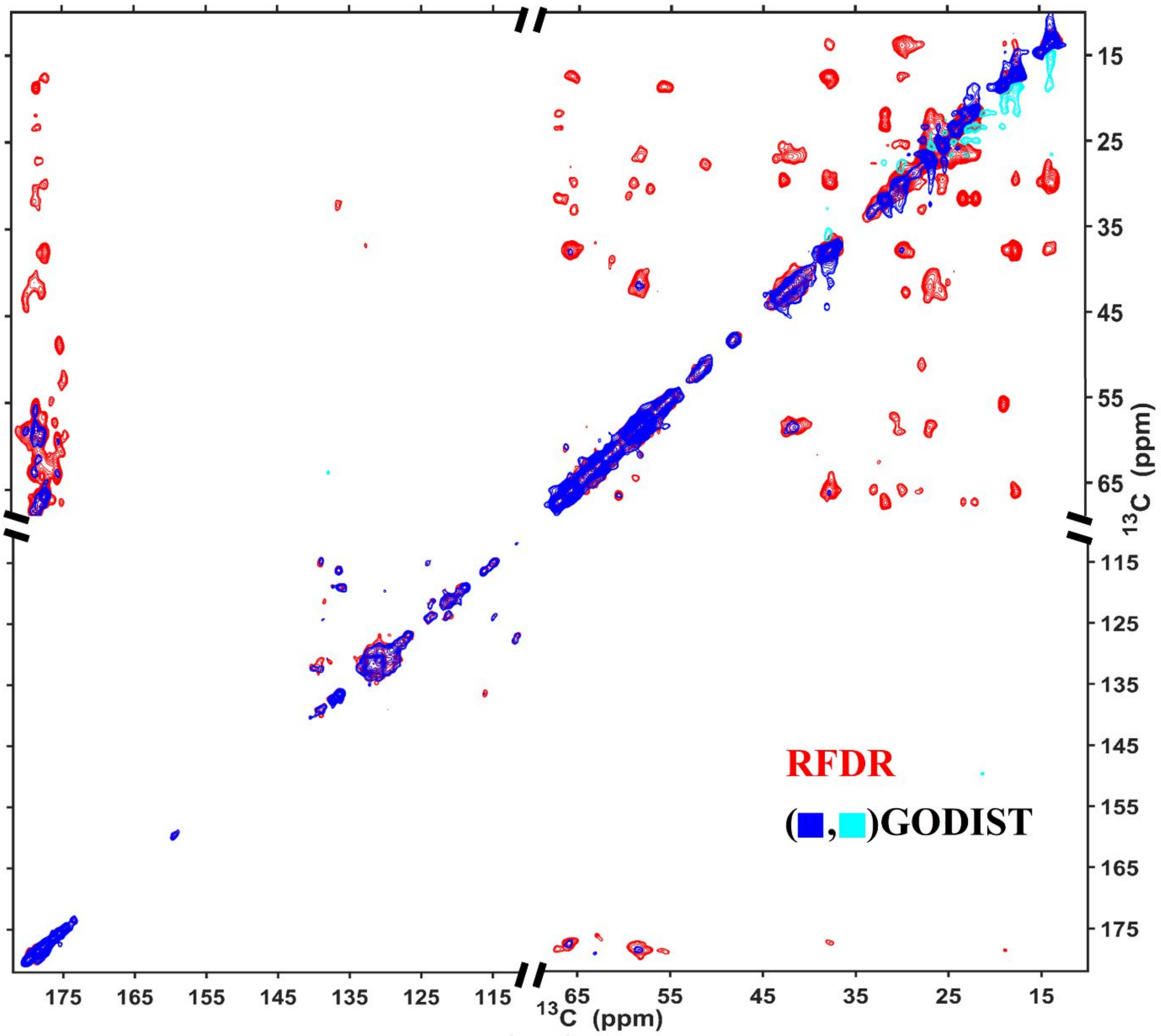


**Figure S6** Characterization of GODIST transfer with the carrier frequency placed in the aromatic region of the spectrum (134 ppm). 2D (H)CC$^{GODIST}$ spectra of Influenza A M2 at 2.304 ms mixing compared with RFDR. GODIST (blue – positive, cyan – negative) and (H)CC$^{RFDR}$ (red) spectra also 2.304 ms mixing. For RFDR, 4.5 μs π-pulses (111.111 kHz rf-field strength) were aplied. The carrier frequency was set to 140 ppm during GODIST and RFDR pulses. Data were acquired at a 600 MHz spectrometer with 55.555 kHz MAS. XY8 phase cycling was used for RFDR. Additional experimental details are provided in the experimental methods section, below.

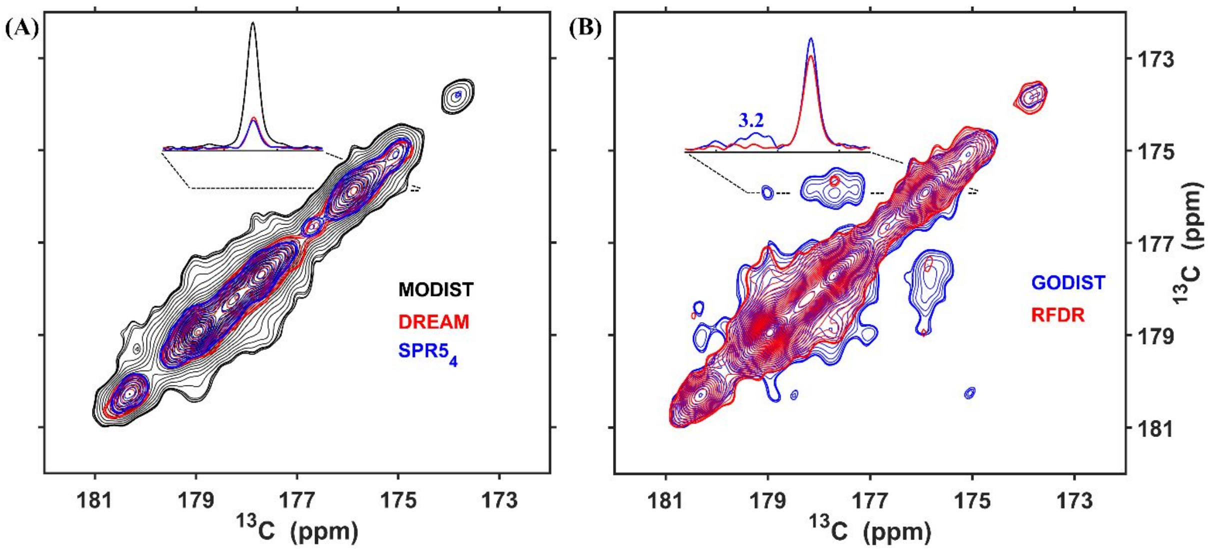


**Figure S7** 2D of Influenza A M2 (H)CC spectra with different recoupling elements. (A) MODIST (black), DREAM (red) and SPR5$_4$ (blue) recoupling elements. The rf-field strengths for MODIST, DREAM and SPR5$_4$ were 27.7 kHz, [13.8:41.6] (ATAN shape) and 34.7 kHz, respectively. (B) GODIST (blue) and RFDR (red). For RFDR, 6 μs (83.333 kHz rf-field strength) π-pulses are applied and XY-16 phase cycling[2] was used for RFDR. For GODIST, 36 μs (27.777 kHz rf-field strength) 2π-pulses were applied. For all three sequences the mixing time was 8.064 ms the carbon carrier frequency was set to 175 ppm. Data was acquired at a 1200 MHz spectrometer with 55.555 kHz MAS.

Table S1. The nineteen carbonyl-carbonyl contacts identified as medium to long range, and the corresponding distances taken from the SH3 crystal structure, PDB code 2NUZ. Peaks were obtained from a 3D (H)COCO(N)H$^{GODIST}$ spectrum of perdeuterated microcrystalline chicken alpha spectrin SH3. The peaks were assigned based on previously determined chemical shifts[3–6].

| $Res_i$-$Res_j$ | r(Å) | $Res_i$-$Res_j$ | r(Å) | $Res_i$-$Res_j$ | r(Å) |
|---|---|---|---|---|---|
| V9-I30 | 4.82 | Y15-M25 | 4.76 | V44-Q50 | 4.84 |
| V9-L31 | 4.97 | R21/E22-G51 | 4.73 | V44-G51 | 5 |
| L10-D29 | 4.93 | E22-F52 | 4.75 | A55-Y57 | 4.75 |
| A11-Y13 | 4.95 | L33-K43 | 4.96 | A55-V58 | 5.22 |
| A11-Y57 | 4.95 | L34-K43 | 4.78 | A56-V58 | 4.93 |
| D14-M25 | 4.76 | W42-F52 | 4.77 | | |
| Y15-T24 | 4.73 | K43-G51 | 4.65 | | |

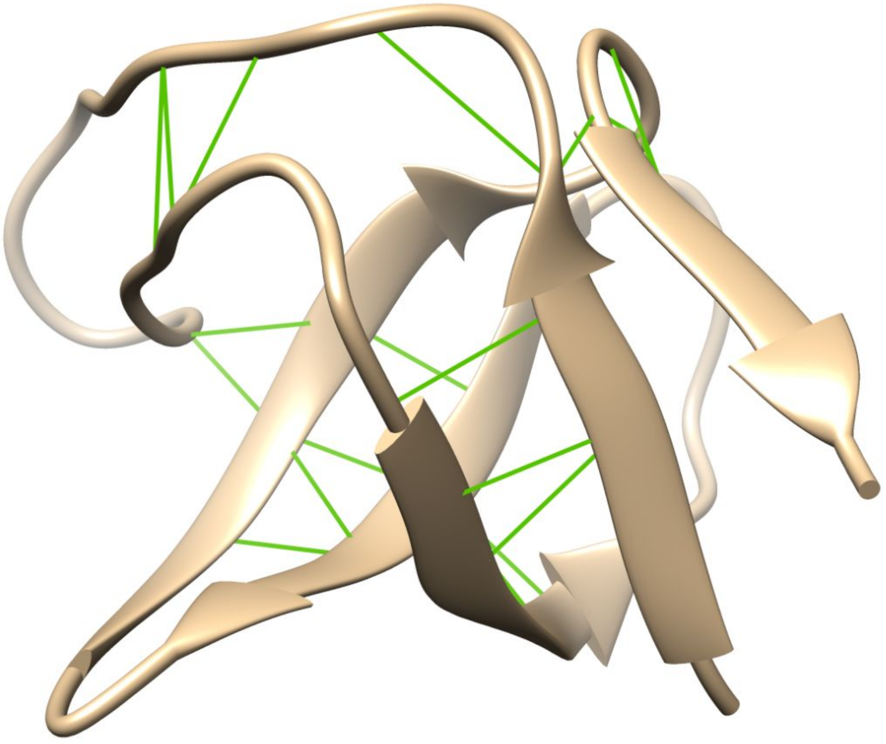

**Figure S8** Visualization of detected CO-CO long-range correlations on the crystal structure of SH3 (PDB code 2NUZ) from a 3D (H)COCO(N)H spectrum of perdeuterated microcrystalline chicken alpha spectrin SH3.

# EXPERIMENTAL METHODS

## Simulations

GODIST simulations were performed using in-house MATLAB scripts with the numerical solution of the equation of motion.[7]

## Sample Preparation

Perdeuterated microcrystalline alpha-spectrin SH3 were prepared according to the published protocols[6,8]. Influenza A M2 protein, residues 18-60, was prepared according to the protocols in the references[9,10] except that 100 mM $Cu^{2+}$ ethylenediaminetetraacetic acid (EDTA) disodium salt was included in the final buffer. Each sample was packed into a Bruker 1.3 mm rotor via centrifugation.

## Solid state NMR spectroscopy

The rf-field power of GODIST pulses was optimized using a single pulse calibration, detected in the 1D (H)C+90° spectrum. The width of the carbon pulse was set to a duration of half a rotor period (for example, 9 µs for 55.555 kHz MAS). The optimal rf-field power was obtained for zero (H)C+90° signal (90°-pulse).

Figures S9 and S10 show 2D (H)CC and 3D (H)COCO(N)H / 3D (H)CO(CO)NH pulse sequences.

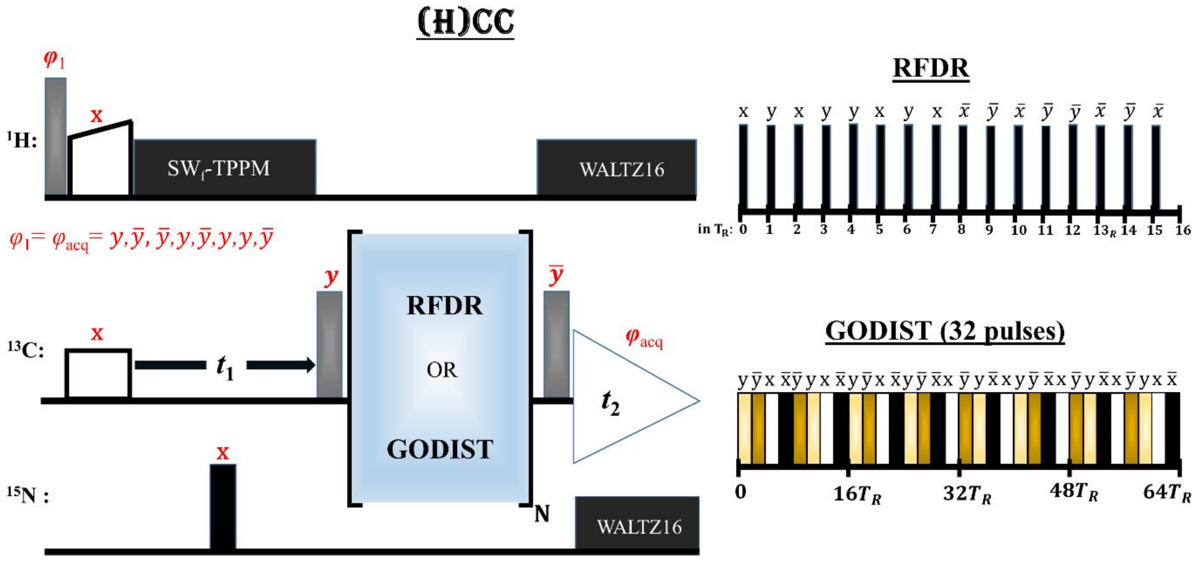


**Figure S9** 2D (H)CC pulse sequence with either RFDR or GODIST recoupling sequences. π/2-pulses are indicated by light, π-pulses by dark grey rectangles. All phase cycling is shown in figure. The ramped CP transfers from proton to carbon are indicated with constant power on the carbon channel and a ramp in power on the proton channel. During the indirect dimension ($t_1$) and acquisition ($t_2$), $SW_f$-TPPM decoupling[11] is applied. A single π-pulse in the middle of $t_1$ and $t_2$ decouples carbon-nitrogen interactions. RFDR: the RFDR elements (eight rotor periods) consists of eight π-pulses every rotor period with XY16 phase cycling.[2,12] GODIST: the GODIST mixing of *N* repeated elements. Each element consists of 64 rotor periods during which 32 2π-pulses are applied with phase cycling: 90°, 270°, 0°, 180°, 270°, 90°, 0°, 180°, 90°, 270°, 0°, 180°, 90°, 270°, 180°, 0°, 270°, 90°, 180°, 0°, 90°, 270°, 180°, 0°, 270°, 90°, 180°, 0°, 270°, 90°, 0°, 180°.

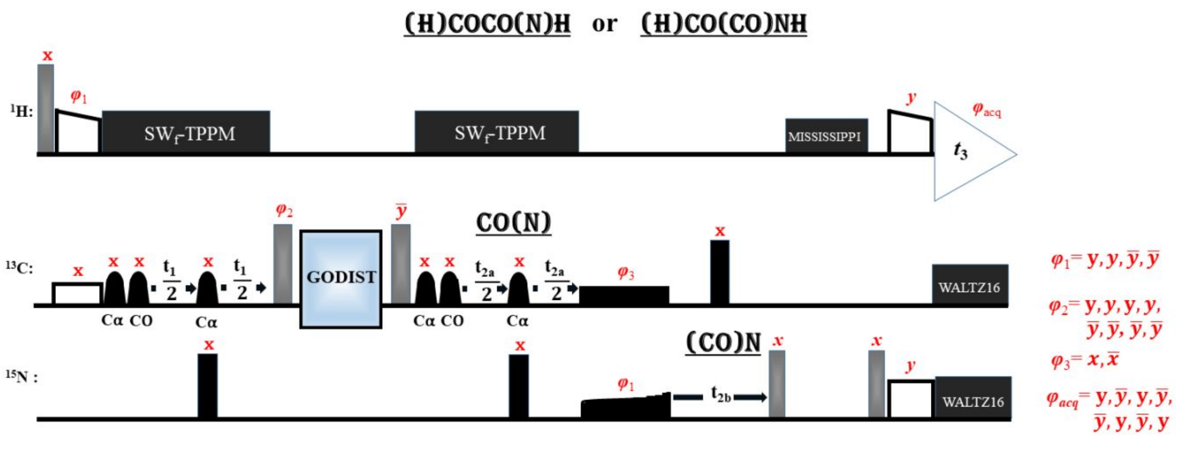


**Figure S10** 3D (H)COCO(N)H or (H)CO(CO)NH sequences, based on extension of the (H)CONH sequence[13]. For (H)COCO(N)H the CO(N) dimention is encoded, and the (CO)N dimention is not ($t_{2B}$) is constant. For (H)CO(CO)NH it is in reverse. The sequence could be extended to four dimensions by sampling both, but this was not implemented here. π/2-pulses are indicated by grey rectangles and π-pulses by black rectangles. REBURP was used for the soft selective pulses[14] on carbon channel to select $^{13}$CO spins. All phase cycling is shown in figure. The ramped CP transfers from proton to carbon as well as from nitrogen to proton are indicated with constant power on the carbon or nitrogen channels and a ramp in power on the proton channel. For carbon→nitrogen CP tranfer, a tangential (ATAN) shape was used. During the inderect dimensions ($t_1$) and ($t_{2a}$ or $t_{2b}$), $SW_f$-TPPM decoupling[11] was applied. A single π-pulse in the middle of $t_1$ and $t_{2a}$ ($t_{2b}$) decouples carbon-nitrogen interactions. Water supression was implemented with the MISSISSIPI sequence.[15] During acquisition, WALTZ16 decoupling[16] was applied on nitrogen and carbon channels. The GODIST recoupling sequence is shown in Figure S9.

*600 MHz:* 2D (H)CC (Figure 2 in the main text, Figures S3-6), 3D (H)COCO(N)H and (H)CO(CO)NH (Figure 3-4 in the main text) experiments were acquired on a Bruker Avance III HD spectrometer operating at 14.1 T (600 MHz $^1$H frequency) using a DVT600W2 BL1.3 mm HXY probe. The experiments were performed at 55.555 kHz MAS, and the temperature of the nitrogen cooling gas set to 245 K with 1000 to 1300 liters per hour. For decoupling of the heteronuclear dipolar interactions $SW_f$-TPPM,[11] was used on the proton channel, and WALTZ-16[16] was used on heteronuclear channels. MISSISSIPPI[15] water suppression was applied for proton detected experiments. In all Figures below: SW – spectral

width; TD – the number of points in the FID; IN_F – an increment time; AQ – the acquisition time. 4 Dummy Scans were used.

*2D (H)CC d-SH3 with GODIST or RFDR*

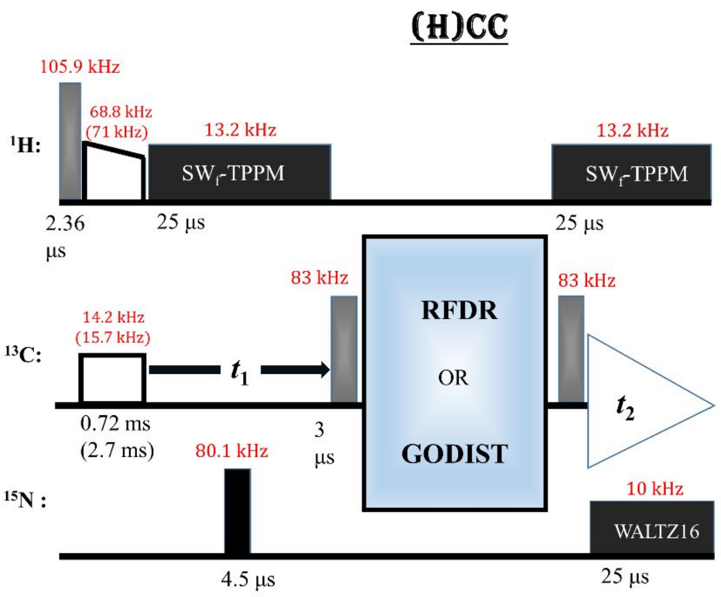


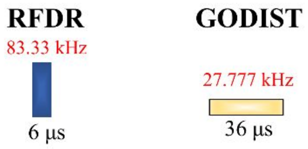


| (H)COC | $^{13}$C | $^{13}$C |
|---|---|---|
| TD | 3072 | 360 |
| SW(ppm) | 553.0965 | 120 |
| IN_F(µs) | | 55.31 |
| AQ(s) | 0.01494 | 0.00999557 |

**CF=173 ppm | Number Scans: 16 | Recycle delay: 2 s**
**Total Time of the experiment: 3.28 hours |**

| (H)C$_\alpha$C | $^{13}$C | $^{13}$C |
|---|---|---|
| TD | 2490 | 560 |
| SW(ppm) | 553.1628 | 200 |
| IN_F(µs) | | 33.19 |
| AQ(s) | 0.01494 | 0.00929 |

**CF=50 ppm | Number Scans: 16 | Recycle delay: 2 s**
**Total Time of the experiment: 5.07 hours |**

**Figure S11** The 2D (H)CC sequence and the experimental parameters used for measurement on perdeuterated microcrystalline SH3. Red numbers represent rf-field strength in kHz. The width of the hard pulses are in µs, while total duration of CP and decoupling are in ms. For H→CO and H→C$_\alpha$ transfers wer acomplished with SPECIFIC-CP[17] with [100%:90%] ramp applied on the proton channel and low rf-field power on the carbon channel (as shown in red in the figure) . CP conditions H→C$_\alpha$ are shown in brackets (). for For ramped CP, the values are shown in the middle of profiles.

*2D (H)CC M2 with GODIST or RFDR*

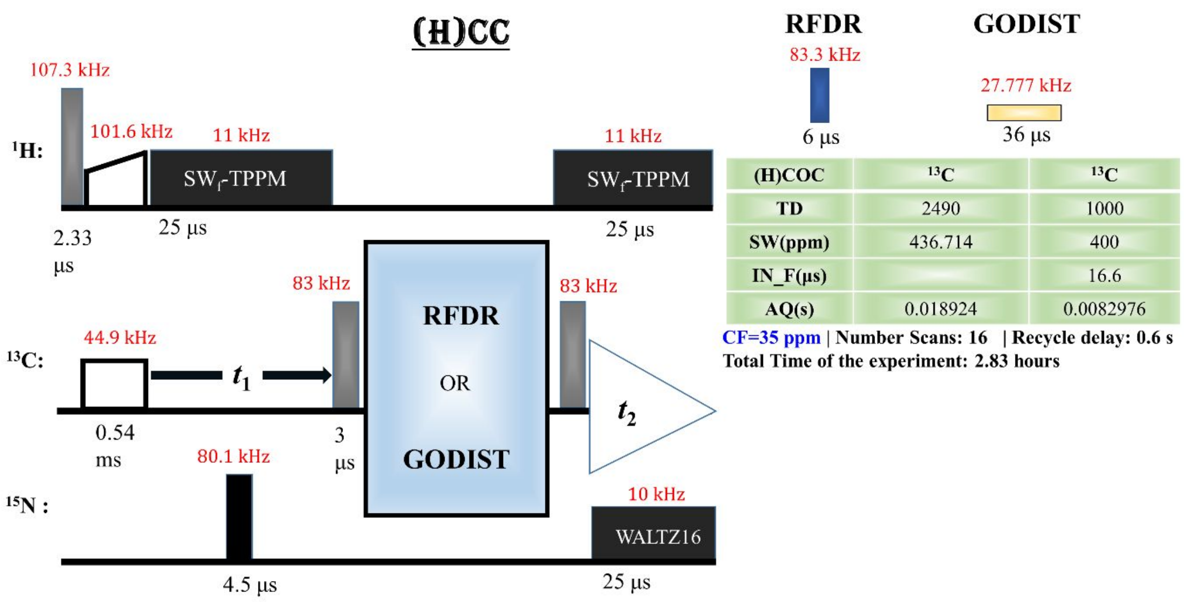


| (H)COC | $^{13}C$ | $^{13}C$ |
|---|---|---|
| TD | 2490 | 1000 |
| SW(ppm) | 436.714 | 400 |
| IN_F(μs) | | 16.6 |
| AQ(s) | 0.018924 | 0.0082976 |

CF=35 ppm | Number Scans: 16 | Recycle delay: 0.6 s
Total Time of the experiment: 2.83 hours

**Figure S12** The 2D (H)CC sequence and the experimental parameters used for measurement on M2. Red numbers represent rf-field strength in kHz. The width of the hard pulses are in μs, while total duration of CP and decoupling are in ms. For H→C transfers CP with [80%:100%] ramp is applied on proton channel. For ramped CP, the values are shown in the middle of profiles.

## *3D (H)COCO(N)H and (H)CO(CO)NH d-SH3 with GODIST*

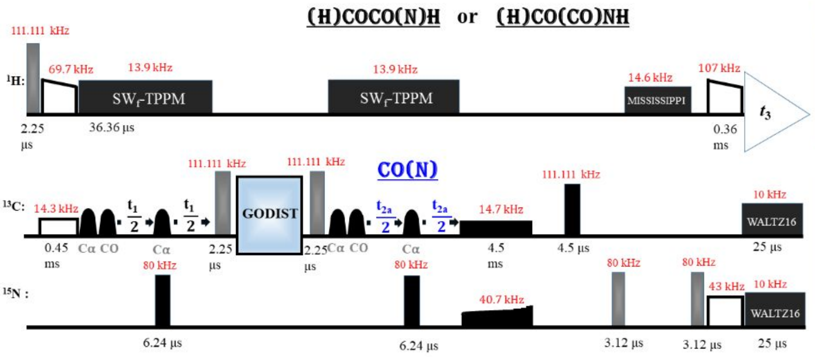


| | $^{1}H$ | $^{13}C$ | $^{13}C$ |
|---|---|---|---|
| TD | 1024 | 150 | 150 |
| SW(ppm) | 46.3664 | 30 | 30 |
| IN_F(μs) | | 221.24 | 221.24 |
| AQ(s) | 0.018432 | 0.0165929 | 0.0165929 |

Number Scans: 16 | Recycle delay: 2 s |
Total Time of the experiment: 9 days

| | $^{1}H$ | $^{13}C$ | $^{15}N$ |
|---|---|---|---|
| TD | 1024 | 160 | 96 |
| SW(ppm) | 46.3664 | 40 | 40 |
| IN_F(μs) | | 165.93 | 411.78 |
| AQ(s) | 0.018432 | 0.0132743 | 0.0197654 |

Number Scans: 8 | Recycle delay: 2 s |
Total Time of the experiment: 3.072 days

**Figure S13** The 3D (H)COCO(N)H and (H)CO(CO)NH sequences and the experimental parameters used for measurement on deuterated SH3. Red numbers represent rf-field strength in kHz. The width of the hard pulses are shown in μs, while total duration of CP and decoupling are in ms. For H→C transfers CP with [100%:90%] ramp was applied on proton channel. For C→N tranfer, ATAN CP with [64%-94%] ramp was used on nitrogen channel. For N→H transfers CP with [100%:80%] ramp was applied on the proton channel. For ramped CP, the values are shown in the middle of profiles. During GODIST pulses, the carrier frequency was set to 185 ppm.

## *3D (H)CO(CO)NH M2 with GODIST*

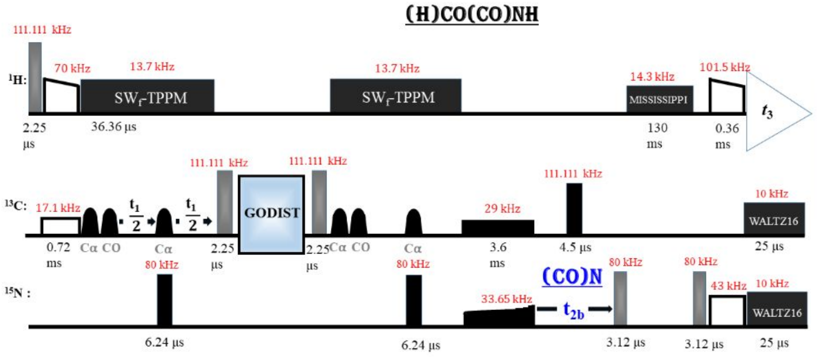


| | $^{1}$H | $^{13}$C | $^{15}$N |
|---|---|---|---|
| TD | 1024 | 146 | 72 |
| SW(ppm) | 46.3664 | 38 | 38 |
| IN_F(µs) | | 174.66 | 433.45 |
| AQ(s) | 0.018432 | 0.0127503 | 0.0156042 |

**Number Scans: 48 | Recycle delay: 2 s |**
**Total Time of the experiment: 12.79 days**

**Figure S14** The 3D (H)CO(CO)NH sequence and the experimental parameters used for measurement on deuterated SH3. Red numbers represent rf-field strength in kHz. Rectangle brackets indicate the minimal and maximal rf-field of ramped CPs. The width of the hard pulses are in µs, while total duration of CP and decoupling are in ms. For H→C transfers CP with [100%:90%] ramp is applied on proton channel. For C→N tranfer, ATAN CP with [64%-94%] ramp was used on nitrogen channel. For N→H transfers CP with [100%:80%] ramp was applied on proton channel. For ramped CP, the values are shown in the middle of profiles. During GODIST pulses, CF is set to 185 ppm.

*1200 MHz:* 2D (H)CC spectra with GODIST, RFDR, DREAM[18,19] and $SPR5_4$[20] recoupling elements (Figure S7) were acquired on a Bruker Avance NEO spectrometer operating at 28.18 T (1200 MHz $^{1}$H frequency) using a 1.3 mm HCN probe. The experiments were performed at 55.555 kHz MAS, the temperature of the nitrogen cooling gas set to 245 K using 1000 liters per hour of flow. For decoupling of the heteronuclear dipolar interactions and water suppression SW$_f$-TPPM,[11] WALTZ-16[16] and MISSISSIPPI[15] were applied. In the Figure below: SW – spectral width; TD – the size of FID; IN_F – an increment time; AQ – the acquisition time. 4 Dummy Scans were used.

## *2D (H)CC M2 with GODIST, RFDR, DREAM or $SPR5_4$*

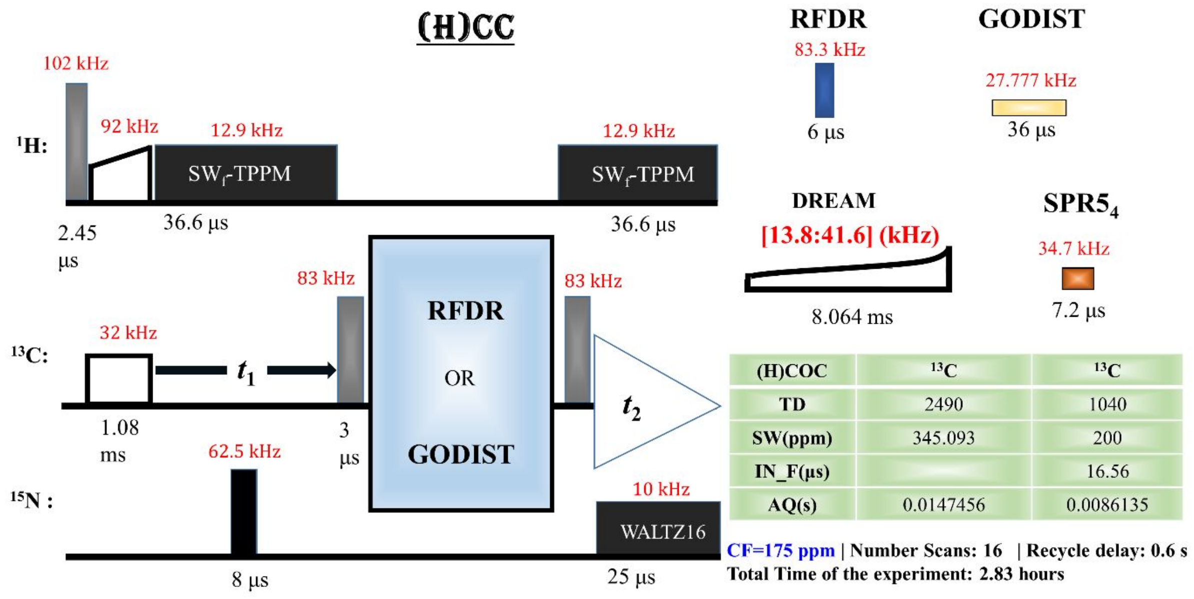


| (H)COC | 13C | 13C |
|---|---|---|
| TD | 2490 | 1040 |
| SW(ppm) | 345.093 | 200 |
| IN_F(µs) | | 16.56 |
| AQ(s) | 0.0147456 | 0.0086135 |

CF=175 ppm | Number Scans: 16 | Recycle delay: 0.6 s
Total Time of the experiment: 2.83 hours

**Figure S15** The 2D (H)CC sequence and the experimental parameters used for measurement on M2. Red numbers represent rf-field strength in kHz. The width of the hard pulses are in µs, while total duration of CP and decoupling are in ms. For H→C transfers CP with [80%:100%] ramp is applied on proton channel. For ramped CP, the values are shown in the middle of profiles. For DREAM the ramped shape (ATAN) was used.

# BRUKER PULSE PROGRAMS

The width of GODIST pulses is automatically calculated using the 'cnst31' parameter (the MAS rate in Hz). Note that there is currently no protection against long acquisitions that occur if this parameter is mistakenly set too low. Use only at your own risk.

## 2D (H)CC GODIST

```
;2D GODIST mixing experiment with carbon detection
;with 15N and 13C decoupling
;Avance II+ version
;parameters:
;p1 : 13C 90 pulse @ plw1
;pl1 : 13C power for 90 pulse

;spnam0 : Ramp90.100
;spoffs0 : 0

;p15 : contact time for H->C CP
;pl20 : 13C power for CP
;sp6 : 1H power for H->C CP
;spnam10 : Ramp100.90
```

```
;spoffs10 : 0

;p3 : 15N 90 pulse @ plw7
;pl3 : 15N power for 90 pulse

;pl5 : GODIST rf-power; 0.5*MAS rate
;l21 : Number of GODIST mixing blocks

;p2 : 1H 90 pulse @ plw2
;pl2 : 1H power for 90 pulse


;cpdprg4 : Water suppression with cwX_pl12 @ 15kHz
;cpdprg5 : Water suppression with cwY_pl12 @ 15kHz
;cpdprg2 : 1H decoupling (sltppm_40pTr41 for 1Hprot, waltz16_pl12 for 2Hprot)
;cpdprg3 : 15N decoupling (waltz16_pl17)

;pl12 : 1H decoupling)
;pl18 : 1H decoupling for (cw_pl18)
;pl13: water suppression
;pl17: 15N decoupling power (waltz16)

;pcpd4 : 33.33 (sltppm 15kHz)
;pcpd2 : 25u (waltz16 10 kHz) - 33.33 (sltppm 15kHz)
;pcpd3 : 25u (waltz16 10 kHz)


;$COMMENT=basic cp experiment, arbitrary contact and decoupling schemes
;$CLASS=Solids
;$DIM=1D
;$TYPE=cross polarisation
;$SUBTYPE=simple 1D
;$OWNER=Bruker
prosol relations=<solids_cp>

#include <Avancesolids.incl>

;cnst20 : RF field achieved at pl13
;cnst21 : on resonance, usually = 0
;cnst22 : positive LG offset
;cnst23 : negative LG offset
;cnst24 : additional LG-offset
;cnst11 : to adjust t=0 for acquisition, if digmod = baseopt
"acqt0=1u*cnst11"
"in0=inf1"
"in30=inf1"

"p11 = ((2s/cnst31))"
"l22=32"

define delay mix
```

```
"mix = (l21*(l22*p11))"

1m
  if "p11   > 100u"    goto Problem
  if "mix   > 300m"    goto Problem
  if "p15   > 15m"  goto Problem
  if "aq    > 45m"    goto Problem
  goto PassParams
Problem, 1m
  print "Parameters not accepted, ending."
  goto HaltAcqu
PassParams, 1m

1 ze
  mix
2 d1 do:f2 do:f3
#include <p15_prot.incl>
#include <aq_prot.incl>
  (p2  pl2 ph1):f2
  (p15:sp1 ph2):f1 (p15:sp0 ph10):f2

  1u cpds2:f2
if "p3*2 > d0" goto RAWEVOL
   (center (d0) (p3*2 ph0 pl3):f3)
if "p3*2 <= d0" goto DECOFF

RAWEVOL, 1u
  d0

DECOFF, 1u do:f2

(p1 pl1 ph4):f1
;;;;;;;;;;;;;cnst19 is used to put carbon carrier frequency during GODIST
1u fq=cnst19(ppm):f1
 ;;;;;;;;;;;;;;; GODIST block starts
3
4 (p11 pl5 ph8^):f1
  lo to 4 times l22
  lo to 3 times l21
;;;;;;;;;;;;;;; GODIST block ends
  1u fq=cnst21(ppm):f1
  1u cpds2:f2
  4m
  (p1 pl1 ph5):f1
  1u cpds3:f3
  go=2 ph31
  1m do:f2     do:f3
  10m mc #0 to 2 F1PH(calph(ph2,+90), caldel(d0,+in0))
HaltAcqu, 1m      ;jump address for protection files
exit                    ;quit
```

ph0= 0
ph1= 1 3 3 1 3 1 1 3
ph10= 0
ph2=  0
ph4= 1
ph5= 3
ph31= 1 3 3 1 3 1 1 3

ph8= 1 3 0 2 3 1 0 2 1 3 0 2 1 3 2 0 3 1 2 0 1 3 2 0 3 1 2 0 3 1 0 2

## 3D (H)COCO(N)H GODIST

; 3D (H)COCO(N)H GODIST
; modified version of 3D (H)CONH developed at CRMN in the group of G. Pintacuda
; Recent notes:
; this version does not require 13C axis inversion

;Avance III version
;parameters:
;p1 : 1H 90 pulse duration
;p3 : 13C 90 pulse duration
;p7 : 15N 90 pulse duration
;d30 : water suppression time (30-100 ms)
;cnst21 : CO offset in ppm (173.7)
;cnst22 : CA offset in ppm (53.7)
;cnst23 : CO/CO offset in ppm (113.7)
;d1 : recycle delay
;d0     : 1/2 of 13CO initial evolution time
;d10    : 1/2 of 13CO initial evolution time
;in0    : 1/2 increment for 13CO evolution
;in10   : 1/2 increment for 13CO evolution
;cpdprg1 : tppm (at pl13) or waltz (at pl13)
;cpdprg4 : cwY (at pl12)
;cpdprg5 : cwX (at pl12)
;pcpd1   : pulse length in decoupling sequence (2xtau_r for tppm, 25us for 10kHz waltz)
;pl1    : power level of 1H hard pulse
;pl12   : power level for 1H decoupling (usually swTPPM at MAS/4 -1 or -2 kHz)
;pl13   : power level of MISSISSIPI water suppression (10-15 kHz)
;spnam1  : 1H shape for 1H->13CO CP (ramp 10-20%)
;spoal1  : N/A
;spoff1 : [ON/RES]
;sp1    : 1H power level during 1H->13CO CP
;spnam10 : 1H shape for 15N->1H(N) CP (ramp 10-20%)
;spoal10  : N/A
;spoff10 : [ON/RES]
;sp10    : 1H power level during 15N->1H CP
;cpdprg2 : 15N decoupling pattern during acq (waltz-16)
;p17     : contact time 15N->1H(N) CP (300-500 us)
;pcpd2   : pulse length in 15N decoupling sequence (25 us)
;pl7    : power level for 15N hard pulse

```
;pl16   : power level for 15N decoupling (corr. to 10 kHz)
;pl2    : power level for 15N hard pulse
;pl20   : 15N power level for 15N->1H CP
;spnam2  : 15N shape for 13CO->15N CP (tan-c100-w10pct)
;sp2    : 15N power level for 13CO->15N CP
;spoal2  : N/A
;spoff2 : [ON/RES]
;p15     : contact time 1H->13CO CP (5 ms)
;p16     : contact time 13CO->15N CP (10 ms)
;p18     : Q3 CO pulse duration
;p19     : Q3 CA pulse duration
;p30        : water suppression duration
;pcpd3   : pulse length in 13C decoupling sequence (25 us)
;cpdprg3 : 13CO/CA decoupling pattern during 15N evol (waltz-16)
;pl3    : power level of 13C hard pulse [REFERENCE]
;pl17   : power level for 13CO/CA decoupling (10 kHz)
;spnam8  : 13C shape for 13CO->15N CP (rectangle)
;sp8     : 13C power for 13CO->15N CP
;spoal8  : N/A
;spoff8 : [ON/RES CO]
;spnam18 : 13CO selective pulse shape (Q3)
;spoal18  : N/A
;spoff18 : [ON/RES CO]
;spnam19 : 13CA selective pulse shape (Q3)
;spoal19  : N/A
;spoff19 : [ON/RES CA]
;spnam28 : 13C shape for 1H->13CO CP (rectangle)
;sp28   : 13C power level for 1H->13CO CP
;spoal28  : N/A
;spoff28 : [ON/RES CO]
;zgoptns : -Dfslg, -Dlacq, or blank


;$COMMENT=Inverse Cp with INEPT CBCA mixing
;$CLASS=Solids
;$DIM=3D
;$TYPE=H detect
;$SUBTYPE=Heteronuclear
;$OWNER=CRMN

#include <Avancesolids.incl>
; Start evolutions from exactly 0
"d0=0.0"
"d10=0.0"

; 1H settings
"spoal1=0.5"   ; default value (irrelevant)
"spoff1=0.0"    ; on-resonance
"spoal10=0.5"  ; default value (irrelevant)
"spoff10=0.0"   ; on-resonance
```

```
; 15N settings
"pcpd2=25"                    ;does not work!
"plw2=plw7"
"plw16=plw2*(pow(p7/25,2))"     ; 15N waltz 10kHz decoupling power level
"spoal2=0.5"   ; default value (irrelevant)
"spoff2=0.0"     ; on-resonance

; 13C settings

;pl5 : GODIST rf-power; 0.5*MAS rate
;l21 : Number of GODIST mixing blocks

"plw17=plw3*(pow(p3/25,2))"     ; 15N waltz 10kHz decoupling power level

"cnst21 = (sfo3-bf3)*1000000/bf3"  ; CO frequency offset (ppm)
"cnst22 = cnst21-(173.7-53.7)"     ; CA frequency offset (ppm)
"cnst23 = cnst21-(173.7-113.7)"    ; the offset half-way CO and CA (ppm)

"p18=3.412/(95.0*bf3/1000000)"    ; 95 ppm bandwidth (safe)
"spw18=plw3*pow((0.5/(p18*0.1515))/(0.25/p3),2)" ;  Q3 power level
"spoal18=0.5"                   ; default value (irrelevant)
"spoff18=0.0"

"p19=3.412/(105.0*bf3/1000000)"      ; 105 ppm bandwidth (safe)
"spw19=plw3*pow((0.5/(p19*0.1515))/(0.25/p3),2)" ;  Q3 power level
"spoal19=0.5"                        ; default value (irrelevant)
"spoff19=bf3*((cnst22-cnst21)/1000000)" ; CA frequency

"spoal8=0.5"                    ; default value (irrelevant)
"spoff8=0.0"                     ; on-resonance

"spoal28=0.5"                    ; default value (irrelevant)
"spoff28=0.0"                     ; on-resonance

;"acqt0=1u*cnst11" ??
"in0=inf1/2"
"in10=inf2/2"

"p11 = ((2s/cnst31))"
"l22=32"

define delay mix
"mix = (l21*(l22*p11))"

;aqseq 312
;aqseq 321

1 ze
  mix
2 d1 do:f2 do:f3
#include <p15_prot.incl>
```

```
#include <aq_prot.incl>
 1u fq=cnst21(bf ppm):f3                          ;go back to the CO frequency
 (p1 pl1 ph3):f1

 (p15:sp28 ph15):f3 (p15:sp1 ph20):f1
 1u  cpds1:f1
 (p19:sp19 ph19):f3  ;CA selective Pi
 1u
 (p18:sp18 ph18):f3   ;CO selective Pi
 1u
 d10
 (center (p7*2 ph0 pl7):f2 (p19:sp19 ph19):f3)  ;CA selective Pi
 d10
(p3 pl3 ph9):f3

;;;;;;;;;;;;;cnst19 is used to put carbon carrier frequency during GODIST
 1u fq=cnst20(ppm):f3
 1u fq=cnst19(ppm):f3
 3m
 1u do:f1
 ;;;;;;;;;;;;;;; GODIST block starts
3
4 (p11 pl5 ph8^):f3
 lo to 4 times l22
 lo to 3 times l21
;;;;;;;;;;;;;;; GODIST block ends
 1u fq=cnst20(ppm):f3
 1u fq=cnst21(bf ppm):f3
 1u cpds1:f1
 3m
 (p3 pl3 ph12):f3

 (p19:sp19 ph19):f3  ;CA selective Pi
 1u
 (p18:sp18 ph18):f3   ;CO selective Pi
 1u
 d0
 (center (p7*2 ph0 pl7):f2 (p19:sp19 ph19):f3)  ;CA selective Pi
 d0
 1u do:f1

 (p16:sp8 ph10):f3 (p16:sp2 ph2):f2
 1u fq=cnst23(bf ppm):f3
(p7 pl7 ph5):f2

(p30*0.25 pl13 ph0):f1
(p30*0.25 pl13 ph1):f1
(p30*0.25 pl13 ph0):f1
(p30*0.25 pl13 ph1):f1

 (p7 pl7 ph6):f2
```

```
 (p17 pl20 ph7):f2 (p17:sp10 ph11):f1

 1u cpds2:f2 cpds3:f3
 go=2 ph31
 1m do:f2 do:f3

 10m mc #0 to 2
 F1PH(calph(ph12, +90), caldel(d0, +in0))   ;13C evolution
 F2PH(calph(ph15, -90), caldel(d10, +in10))   ;13C evolution


HaltAcqu, 1m      ;jump address for protection files
exit              ;quit

ph0=0
ph1=1
ph3=0
ph20=1 1 3 3
ph15=0
ph10=0 2
ph2=1 1 3 3 1 1 3 3
ph5=0
ph12= 3
ph9=1 1 1 1 3 3 3 3
ph6=0
ph18=0
ph19=0
ph7=1
ph11=1
ph31=1 3 1 3 3 1 3 1

ph8= 1 3 0 2 3 1 0 2 1 3 0 2 1 3 2 0 3 1 2 0 1 3 2 0 3 1 2 0 3 1 0 2
```

## 3D (H)CO(CO)NH GODIST

```
; 3D (H)CO(CO)NH GODIST
; modified version of 3D (H)CONH developed at CRMN in the group of G. Pintacuda
; Recent notes:
; this version does not require 13C axis inversion

;Avance III version
;parameters:
;p1 : 1H 90 pulse duration
;p3 : 13C 90 pulse duration
;p7 : 15N 90 pulse duration
;d30 : water suppression time (30-100 ms)
;cnst21 : CO offset in ppm (173.7)
;cnst22 : CA offset in ppm (53.7)
;cnst23 : CO/CO offset in ppm (113.7)
;d1 : recycle delay
;d0     : 1 of 15N initial evolution time
```

;d10    : 1/2 of 13CO initial evolution time
;in0    : 1 increment for 15N evolution
;in10   : 1/2 increment for 13CO evolution
;cpdprg1 : tppm (at pl13) or waltz (at pl13)
;cpdprg4 : cwY (at pl12)
;cpdprg5 : cwX (at pl12)
;pcpd1   : pulse length in decoupling sequence (2xtau_r for tppm, 25us for 10kHz waltz)
;pl1    : power level of 1H hard pulse
;pl12   : power level for 1H decoupling (usually swTPPM at MAS/4 -1 or -2 kHz)
;pl13   : power level of MISSISSIPI water suppression (10-15 kHz)
;spnam1  : 1H shape for 1H->13CO CP (ramp 10-20%)
;spoal1  : N/A
;spoff1 : [ON/RES]
;sp1    : 1H power level during 1H->13CO CP
;spnam10 : 1H shape for 15N->1H(N) CP (ramp 10-20%)
;spoal10  : N/A
;spoff10 : [ON/RES]
;sp10    : 1H power level during 15N->1H CP
;cpdprg2 : 15N decoupling pattern during acq (waltz-16)
;p17     : contact time 15N->1H(N) CP (300-500 us)
;pcpd2   : pulse length in 15N decoupling sequence (25 us)
;pl7    : power level for 15N hard pulse
;pl16   : power level for 15N decoupling (corr. to 10 kHz)
;pl2    : power level for 15N hard pulse
;pl20   : 15N power level for 15N->1H CP
;spnam2  : 15N shape for 13CO->15N CP (tan-c100-w10pct)
;sp2    : 15N power level for 13CO->15N CP
;spoal2  : N/A
;spoff2 : [ON/RES]
;p15     : contact time 1H->13CO CP (5 ms)
;p16     : contact time 13CO->15N CP (10 ms)
;p18     : Q3 CO pulse duration
;p19     : Q3 CA pulse duration
;p30        : water suppression duration
;pcpd3   : pulse length in 13C decoupling sequence (25 us)
;cpdprg3 : 13CO/CA decoupling pattern during 15N evol (waltz-16)
;pl3    : power level of 13C hard pulse [REFERENCE]
;pl17   : power level for 13CO/CA decoupling (10 kHz)
;spnam8  : 13C shape for 13CO->15N CP (rectangle)
;sp8     : 13C power for 13CO->15N CP
;spoal8  : N/A
;spoff8 : [ON/RES CO]
;spnam18 : 13CO selective pulse shape (Q3)
;spoal18  : N/A
;spoff18 : [ON/RES CO]
;spnam19 : 13CA selective pulse shape (Q3)
;spoal19  : N/A
;spoff19 : [ON/RES CA]
;spnam28 : 13C shape for 1H->13CO CP (rectangle)
;sp28   : 13C power level for 1H->13CO CP
;spoal28  : N/A

```
;spoff28 : [ON/RES CO]
;zgoptns : -Dfslg, -Dlacq, or blank

;pl5 : GODIST rf-power; 0.5*MAS rate
;l21 : Number of GODIST mixing blocks

;$COMMENT=Inverse Cp with INEPT CBCA mixing
;$CLASS=Solids
;$DIM=3D
;$TYPE=H detect
;$SUBTYPE=Heteronuclear
;$OWNER=CRMN

#include <Avancesolids.incl>

; Start evolutions from exactly 0
"d0=0.0"
"d10=0.0"

; 1H settings
"spoal1=0.5"   ; default value (irrelevant)
"spoff1=0.0"    ; on-resonance
"spoal10=0.5"  ; default value (irrelevant)
"spoff10=0.0"   ; on-resonance

; 15N settings
"pcpd2=25"                    ;does not work!
"plw2=plw7"
"plw16=plw2*(pow(p7/25,2))"     ; 15N waltz 10kHz decoupling power level
"spoal2=0.5"   ; default value (irrelevant)
"spoff2=0.0"     ; on-resonance

; 13C settings
"plw17=plw3*(pow(p3/25,2))"     ; 15N waltz 10kHz decoupling power level

"cnst21 = (sfo3-bf3)*1000000/bf3"  ; CO frequency offset (ppm)
"cnst22 = cnst21-(173.7-53.7)"     ; CA frequency offset (ppm)
"cnst23 = cnst21-(173.7-113.7)"    ; the offset half-way CO and CA (ppm)

"p18=3.412/(95.0*bf3/1000000)"    ; 95 ppm bandwidth (safe)
"spw18=plw3*pow((0.5/(p18*0.1515))/(0.25/p3),2)" ;  Q3 power level
"spoal18=0.5"                   ; default value (irrelevant)
"spoff18=0.0"

"p19=3.412/(105.0*bf3/1000000)"      ; 105 ppm bandwidth (safe)
"spw19=plw3*pow((0.5/(p19*0.1515))/(0.25/p3),2)" ;  Q3 power level
"spoal19=0.5"                        ; default value (irrelevant)
"spoff19=bf3*((cnst22-cnst21)/1000000)" ; CA frequency

"spoal8=0.5"                    ; default value (irrelevant)
"spoff8=0.0"                     ; on-resonance
```

```
"spoal28=0.5"                    ; default value (irrelevant)
"spoff28=0.0"                    ; on-resonance

;"acqt0=1u*cnst11" ??
"in0=inf1"
"in10=inf2/2"

"p11 = ((2s/cnst31))"

"l22=32"

define delay mix
"mix = (l21*(l22*p11))"

;aqseq 312
;aqseq 321

1m
  if "mix   > 100m"    goto Problem
  if "p15   > 15m"  goto Problem
  if "aq    > 45m"    goto Problem
  goto PassParams
Problem, 1m
  print "Parameters not accepted, ending."
  goto HaltAcqu
PassParams, 1m

1 ze
  mix
2 d1 do:f2 do:f3
#include <p15_prot.incl>
#include <aq_prot.incl>
  1u fq=cnst21(bf ppm):f3                         ;go back to the CO frequency
  (p1 pl1 ph3):f1

  (p15:sp28 ph15):f3 (p15:sp1 ph20):f1
  1u  cpds1:f1
  (p19:sp19 ph19):f3  ;CA selective Pi
  1u
  (p18:sp18 ph18):f3   ;CO selective Pi
  1u
  d10
  (center (p7*2 ph0 pl7):f2 (p19:sp19 ph19):f3)  ;CA selective Pi
  d10
 (p3 pl3 ph9):f3
  3m
  1u do:f1

;;;;;;;;;;;;;cnst19 is used to put carbon carrier frequency during BASES
 1u fq=cnst20(ppm):f3
```

```
 1u fq=cnst19(ppm):f3
 ;;;;;;;;;;;;;;; GODIST block starts
3
4 (p11 pl5 ph8^):f3
  lo to 4 times l22
  lo to 3 times l21
;;;;;;;;;;;;;;; GODIST block ends
  1u fq=cnst20(ppm):f3
  1u fq=cnst21(bf ppm):f3                    ;go back to the CO frequency
  1u  cpds1:f1
  3m
  (p3 pl3 ph12):f3

  (p19:sp19 ph19):f3  ;CA selective Pi
  1u
  (p18:sp18 ph18):f3   ;CO selective Pi
  1u
  (center (p7*2 ph0 pl7):f2 (p19:sp19 ph19):f3)  ;CA selective Pi
  1u do:f1

  (p16:sp8 ph10):f3 (p16:sp2 ph2):f2
  1u fq=cnst23(bf ppm):f3
  1u cpds1:f1 cpds3:f3
  d0
  1u do:f1 do:f3
  (p7 pl7 ph5):f2

 (p30*0.25 pl13 ph0):f1
 (p30*0.25 pl13 ph1):f1
 (p30*0.25 pl13 ph0):f1
 (p30*0.25 pl13 ph1):f1

  (p7 pl7 ph6):f2
  (p17 pl20 ph7):f2 (p17:sp10 ph11):f1

  1u cpds2:f2 cpds3:f3
  go=2 ph31
  1m do:f2 do:f3

  10m mc #0 to 2
  F1PH(calph(ph2, +90), caldel(d0, +in0)) ;15N
  F2PH(calph(ph15, -90), caldel(d10, +in10))   ;13C evolution


HaltAcqu, 1m      ;jump address for protection files
exit                   ;quit

ph0=0
ph1=1
ph3=0
ph20=1 1 3 3
```

ph15=0
ph10=0 2
ph2=1 1 3 3 3 3 1 1
ph5=0
ph12= 3
ph9=1
ph6=0
ph18=0
ph19=0
ph7=1
ph11=1
ph31=1 3 1 3 3 1 3 1

ph8= 1 3 0 2 3 1 0 2 1 3 0 2 1 3 2 0 3 1 2 0 1 3 2 0 3 1 2 0 3 1 0 2

## 3D (H)CCH GODIST

;1H-detected (H)CCH GODIST experiment
;with 15N and 13C decoupling

;Avance III+ version
;parameters:
;p1 : 1H 90 pulse @ plw1
;pl1 : 1H power for 90 pulse

;p15 : contact time for H->C CP
;sp0 : 1H power for H->C CP
;sp10 : 1H power for C->H CP
;spnam0 : Ramp90.100
;spoffs0 : 0

;d0 : 13C initial evolution time
;d10 : 13C initial evolution time
;in0 : increment for 13CO evolution
;in10 : increment for 13CO evolution

;p7 : 15N 90 pulse @ plw7
;pl7 : 15N power for 90 pulse

; 13C settings

;pl5 : GODIST rf-power; 0.5*MAS rate
;l21 : Number of GODIST mixing blocks

;p3 : 13C 90 pulse @ plw3
;pl3 : 13C power for 90 pulse

;cpdprg1 : 1H decoupling (sltppm_40pTr41 for 1Hprot, waltz16_pl12 for 2Hprot)
;cpdprg4 : Water suppression with cwX_pl12 @ 15kHz
;cpdprg5 : Water suppression with cwY_pl12 @ 15kHz
;cpdprg2 : 15N decoupling (waltz16_pl16)

```
;cpdprg3 : 13C decoupling (waltz16_pl17)

;pl12 : 1H decoupling power for (tppm/waltz16)
;pl18 : 1H decoupling for (cw_pl18)
;pl13: water suppression
;pl16: 15N decoupling power (waltz16)
;pl17: 13C decoupling power (waltz16)

;pcpd1 : 25u (waltz16 10 kHz) - 33.33 (sltppm 15kHz)
;pcpd4 : 33.33 (sltppm 15kHz)
;pcpd2 : 25u (waltz16 10 kHz)
;pcpd3 : 25u (waltz16 10 kHz)


;$COMMENT=basic cp experiment, arbitrary contact and decoupling schemes
;$CLASS=Solids
;$DIM=1D
;$TYPE=cross polarisation
;$SUBTYPE=simple 1D
;$OWNER=Bruker
prosol relations=<solids_cp>

#include <Avancesolids.incl>

;cnst20 : RF field achieved at pl13
;cnst21 : on resonance, usually = 0
;cnst22 : positive LG offset
;cnst23 : negative LG offset
;cnst24 : additional LG-offset
;cnst11 : to adjust t=0 for acquisition, if digmod = baseopt
"acqt0=1u*cnst11"
"in0=inf1"
"in10=inf2"

"p11 = ((2s/cnst31))"

"l22=32"

define delay mix
"mix = (l21*(l22*p11))"

1 ze
  mix
2 d1 do:f2 do:f3
#include <p15_prot.incl>
#include <aq_prot.incl>

  (p1  pl1 ph3):f1
  (p15 pl20 ph2):f3 (p15:sp0 ph10):f1

  1u cpds1:f1
```

```
if "p7*2 > d10" goto RAWEVOL
   (center (d10) (p7*2 ph0 pl7):f2)
if "p7*2 <= d10" goto DECOFF

RAWEVOL, 1u
  d10
DECOFF,  1u
  (p3 pl3 ph9):f3
  1u fq=cnst19(ppm):f3
  3m
  1u do:f1
 ;;;;;;;;;;;;;;; GODIST block starts
3
4 (p11 pl5 ph8^):f3
  lo to 4 times l22
  lo to 3 times l21
;;;;;;;;;;;;;;; GODIST block ends
  1u fq=cnst20(ppm):f3
  1u cpds1:f1
  3m
  (p3 pl3 ph12):f3

  1u cpds1:f1

if "p7*2 > d0" goto RAWEVOL1
   (center (d0) (p7*2 ph0 pl7):f2)
if "p7*2 <= d0" goto DECOFF1

RAWEVOL1, 1u
  d0
DECOFF1,  1u do:f1

;;;;;;;;;;;;;;; water suppression block starts
  (p3 pl3 ph5):f3
  (p30*0.25  pl13 ph0):f1
  (p30*0.25  pl13 ph1):f1
  (p30*0.25  pl13 ph0):f1
  (p30*0.25  pl13 ph1):f1

  (p3 pl3 ph6):f3
;;;;;;;;;;;;;;; water suppression block ends

  (p17 pl20 ph7):f3 (p17:sp10 ph11):f1

  1u cpds2:f2 cpds3:f3
  go=2 ph31
  1m do:f2 do:f3

  10m mc #0 to 2
  F1PH(calph(ph12, +90), caldel(d0, +in0))   ;13C evolution
```

```
 F2PH(calph(ph2, -90), caldel(d10, +in10))   ;13C evolution

HaltAcqu, 1m      ;jump address for protection files
exit              ;quit

ph0=0
ph1=1
ph3  = 1 1 3 3
ph10 = 0
ph2 =  1
ph5 =  0
ph6 =  0 2
ph7 =  1
ph9=0
ph12 = 2
ph11 = 1 1 1 1
ph31 = 1 3 3 1
ph8= 1 3 0 2 3 1 0 2 1 3 0 2 1 3 2 0 3 1 2 0 1 3 2 0 3 1 2 0 3 1 0 2
```

# REFERENCE


(1) Bak, M.; Rasmussen, J. T.; Nielsen, N. C. SIMPSON: A General Simulation Program for Solid-State NMR Spectroscopy. *J. Magn. Reson.* **2000**, *147* (2), 296–330. https://doi.org/10.1006/jmre.2000.2179.

(2) Gullion, T.; Baker, D. B.; Conradi, M. S. New, Compensated Carr-Purcell Sequences. *J. Magn. Reson. 1969* **1990**, *89* (3), 479–484. https://doi.org/10.1016/0022-2364(90)90331-3.

(3) Pauli, J.; Baldus, M.; van Rossum, B.; de Groot, H.; Oschkinat, H. Backbone and Side-Chain 13C and 15N Signal Assignments of the α-Spectrin SH3 Domain by Magic Angle Spinning Solid-State NMR at 17.6 Tesla. *ChemBioChem* **2001**, *2* (4), 272–281. https://doi.org/10.1002/1439-7633(20010401)2:4<272::AID-CBIC272>3.0.CO;2-2.

(4) van Rossum, B.-J.; Castellani, F.; Pauli, J.; Rehbein, K.; Hollander, J.; de Groot, H. J. M.; Oschkinat, H. Assignment of Amide Proton Signals by Combined Evaluation of HN, NN and HNCA MAS-NMR Correlation Spectra. *J. Biomol. NMR* **2003**, *25* (3), 217–223. https://doi.org/10.1023/A:1022819921584.

(5) Linser, R.; Fink, U.; Reif, B. Assignment of Dynamic Regions in Biological Solids Enabled by Spin-State Selective NMR Experiments. *J. Am. Chem. Soc.* **2010**, *132* (26), 8891–8893. https://doi.org/10.1021/ja102612m.

(6) Chevelkov, V.; Rehbein, K.; Diehl, A.; Reif, B. Ultrahigh Resolution in Proton Solid-State NMR Spectroscopy at High Levels of Deuteration. *Angew. Chem. Int. Ed.* **2006**, *45* (23), 3878–3881. https://doi.org/10.1002/anie.200600328.

(7) Nimerovsky, E.; Goldbourt, A. Insights into the Spin Dynamics of a Large Anisotropy Spin Subjected to Long-Pulse Irradiation under a Modified REDOR Experiment. *J. Magn. Reson.* **2012**, *225*, 130–141. https://doi.org/10.1016/j.jmr.2012.09.015.

(8) Xue, K.; Mühlbauer, M.; Mamone, S.; Sarkar, R.; Reif, B. Accurate Determination of 1H-15N Dipolar Couplings Using Inaccurate Settings of the Magic Angle in Solid-State NMR Spectroscopy. *Angew. Chem. Int. Ed.* **2019**, *58* (13), 4286–4290. https://doi.org/10.1002/anie.201814314.

(9) Schnell, J. R.; Chou, J. J. Structure and Mechanism of the M2 Proton Channel of Influenza A Virus. *Nature* **2008**, *451* (7178), 591–595. https://doi.org/10.1038/nature06531.

(10) Andreas, L. B.; Eddy, M. T.; Pielak, R. M.; Chou, J.; Griffin, R. G. Magic Angle Spinning NMR Investigation of Influenza A M218−60: Support for an Allosteric Mechanism of Inhibition. *J. Am. Chem. Soc.* **2010**, *132* (32), 10958–10960. https://doi.org/10.1021/ja101537p.
(11) Thakur, R. S.; Kurur, N. D.; Madhu, P. K. Swept-Frequency Two-Pulse Phase Modulation for Heteronuclear Dipolar Decoupling in Solid-State NMR. *Chem. Phys. Lett.* **2006**, *426* (4), 459–463. https://doi.org/10.1016/j.cplett.2006.06.007.
(12) Bennett, A. E.; Rienstra, C. M.; Griffiths, J. M.; Zhen, W.; Lansbury, P. T.; Griffin, R. G. Homonuclear Radio Frequency-Driven Recoupling in Rotating Solids. *J. Chem. Phys.* **1998**, *108* (22), 9463–9479. https://doi.org/10.1063/1.476420.
(13) Barbet-Massin, E.; Pell, A. J.; Retel, J. S.; Andreas, L. B.; Jaudzems, K.; Franks, W. T.; Nieuwkoop, A. J.; Hiller, M.; Higman, V.; Guerry, P.; Bertarello, A.; Knight, M. J.; Felletti, M.; Le Marchand, T.; Kotelovica, S.; Akopjana, I.; Tars, K.; Stoppini, M.; Bellotti, V.; Bolognesi, M.; Ricagno, S.; Chou, J. J.; Griffin, R. G.; Oschkinat, H.; Lesage, A.; Emsley, L.; Herrmann, T.; Pintacuda, G. Rapid Proton-Detected NMR Assignment for Proteins with Fast Magic Angle Spinning. *J. Am. Chem. Soc.* **2014**, *136* (35), 12489–12497. https://doi.org/10.1021/ja507382j.
(14) Li, Y.; Wylie, B. J.; Rienstra, C. M. Selective Refocusing Pulses in Magic-Angle Spinning NMR: Characterization and Applications to Multi-Dimensional Protein Spectroscopy. *J. Magn. Reson.* **2006**, *179* (2), 206–216. https://doi.org/10.1016/j.jmr.2005.12.003.
(15) Zhou, D. H.; Rienstra, C. M. High-Performance Solvent Suppression for Proton Detected Solid-State NMR. *J. Magn. Reson.* **2008**, *192* (1), 167–172. https://doi.org/10.1016/j.jmr.2008.01.012.
(16) Shaka, A. J.; Keeler, J.; Frenkiel, T.; Freeman, R. An Improved Sequence for Broadband Decoupling: WALTZ-16. *J. Magn. Reson. 1969* **1983**, *52* (2), 335–338. https://doi.org/10.1016/0022-2364(83)90207-X.
(17) Laage, S.; Marchetti, A.; Sein, J.; Pierattelli, R.; Sass, H. J.; Grzesiek, S.; Lesage, A.; Pintacuda, G.; Emsley, L. Band-Selective 1H−13C Cross-Polarization in Fast Magic Angle Spinning Solid-State NMR Spectroscopy. *J. Am. Chem. Soc.* **2008**, *130* (51), 17216–17217. https://doi.org/10.1021/ja805926d.
(18) Verel, R.; Baldus, M.; Ernst, M.; Meier, B. H. A Homonuclear Spin-Pair Filter for Solid-State NMR Based on Adiabatic-Passage Techniques. *Chem. Phys. Lett.* **1998**, *287* (3), 421–428. https://doi.org/10.1016/S0009-2614(98)00172-9.
(19) Verel, R.; Ernst, M.; Meier, B. H. Adiabatic Dipolar Recoupling in Solid-State NMR: The DREAM Scheme. *J. Magn. Reson.* **2001**, *150* (1), 81–99. https://doi.org/10.1006/jmre.2001.2310.
(20) Zhang, Z.; Oss, A.; Org, M.-L.; Samoson, A.; Li, M.; Tan, H.; Su, Y.; Yang, J. Selectively Enhanced 1H–1H Correlations in Proton-Detected Solid-State NMR under Ultrafast MAS Conditions. *J. Phys. Chem. Lett.* **2020**, *11* (19), 8077–8083. https://doi.org/10.1021/acs.jpclett.0c02412.
